# Preferential Localization of Human Origins of DNA Replication at the 5′-Ends of Expressed Genes and at Evolutionarily Conserved DNA Sequences

**DOI:** 10.1371/journal.pone.0017308

**Published:** 2011-05-13

**Authors:** Manuel S. Valenzuela, Yidong Chen, Sean Davis, Fan Yang, Robert L. Walker, Sven Bilke, John Lueders, Melvenia M. Martin, Mirit I. Aladjem, Pierre P. Massion, Paul S. Meltzer

**Affiliations:** 1 Genetics Branch, Center for Cancer Research, National Cancer Institute, National Institutes of Health, Bethesda, Maryland, United States of America; 2 Department of Biochemistry and Cancer Biology, Meharry Medical College, Nashville, Tennessee, United States of America; 3 Laboratory of Molecular Pharmacology, Center for Cancer Research, National Cancer Institute, National Institutes of Health, Bethesda, Maryland, United States of America; 4 Division of Allergy, Pulmonary and Critical Care Medicine, Vanderbilt Ingram Cancer Center, Vanderbilt University, Nashville, Tennessee, United States of America; Tulane University Health Sciences Center, United States of America

## Abstract

**Background:**

Replication of mammalian genomes requires the activation of thousands of origins which are both spatially and temporally regulated by as yet unknown mechanisms. At the most fundamental level, our knowledge about the distribution pattern of origins in each of the chromosomes, among different cell types, and whether the physiological state of the cells alters this distribution is at present very limited.

**Methodology/Principal Findings:**

We have used standard λ-exonuclease resistant nascent DNA preparations in the size range of 0.7–1.5 kb obtained from the breast cancer cell line MCF–7 hybridized to a custom tiling array containing 50–60 nt probes evenly distributed among genic and non-genic regions covering about 1% of the human genome. A similar DNA preparation was used for high-throughput DNA sequencing. Array experiments were also performed with DNA obtained from BT-474 and H520 cell lines. By determining the sites showing nascent DNA enrichment, we have localized several thousand origins of DNA replication. Our major findings are: (a) both array and DNA sequencing assay methods produced essentially the same origin distribution profile; (b) origin distribution is largely conserved (>70%) in all cell lines tested; (c) origins are enriched at the 5′ends of expressed genes and at evolutionarily conserved intergenic sequences; and (d) ChIP on chip experiments in MCF-7 showed an enrichment of H3K4Me3 and RNA Polymerase II chromatin binding sites at origins of DNA replication.

**Conclusions/Significance:**

Our results suggest that the program for origin activation is largely conserved among different cell types. Also, our work supports recent studies connecting transcription initiation with replication, and in addition suggests that evolutionarily conserved intergenic sequences have the potential to participate in origin selection. Overall, our observations suggest that replication origin selection is a stochastic process significantly dependent upon local accessibility to replication factors.

## Introduction

Initiation of DNA replication is a critical step in the regulation of cell proliferation. The replicon model proposed 46 years ago [1] has served as a good paradigm for our understanding of the initiation step of DNA replication. According to this model, the origin, and adjacent DNA sequences whose replication depend on it, define an independent unit of replication, or replicon. The initiation step relies on the interaction of trans-acting factors (initiators) with cis-acting DNA sequences (replicators or origins). Based on studies on the single replicon present in E. coli, the role of the initiator protein(s) has been expanded to mark the position of the origin, as well as to serve as a recruitment factor that facilitates the opening of the DNA helix, a step required for the initiation of DNA synthesis [2]. Most of our current understanding about the initiation step in eukaryotic DNA replication is based on the wealth of experimental information obtained in both the budding yeast and frog embryos. In the budding yeast, origins are defined by the presence of a small (10–15 bp) conserved DNA sequence, named autonomous replication consensus sequence (ACS), harboring the autonomous replicating sequence (ARS) motif. Regardless of their source, DNA sequences harboring the ARS motif, can promote DNA replication in yeast [3], [4]. A genome wide functional analysis of the distribution of replication origins in budding yeast has shown significant agreement with a computational analysis based solely on the distribution of ARS-related motifs in the yeast genome[4], [5]. These results strongly indicate that in budding yeast, specific DNA sequences dictate the position of the initiation step of DNA replication. At the opposite end of the spectrum, in frog and fly embryos, DNA replication appears to initiate randomly along the genome. Moreover, any DNA sequence, regardless of its source and composition can replicate in these systems, arguing that no specific DNA sequence is required to initiate DNA replication [6]. In metazoans, the temporal regulation of regional initiation of DNA replication and the identification of defined origins of DNA replication which can function ectopically have been presented as arguments for the occurrence of specific DNA sequences at origins [7]. However, to date a specific DNA sequence has not yet been identified, although some degenerate sequences and motifs have been proposed [8], [9]. There is ample evidence suggesting that the number of potential mammalian origins exceeds what is required to duplicate the whole genome, but the distribution of potential origins along the chromosomes and the manner they are activated are still unclear [7], [10], [11].

In this study we have used a DNA microarray-based nascent strand abundance assay, and high-throughput DNA sequencing to determine the distribution of putative origins of DNA replication along selected regions of human chromosomes covering 1% of the human genome. Data from three different cell lines indicate that potential origins are closely spaced (3–5 kb) and that their positioning is largely conserved. More interestingly, our results indicate that origins are not randomly distributed but that are enriched at the 5′-ends of expressed genes as well as at the locations of intergenic conserved sequences. The association of origin positioning with gene expression was further investigated in MCF-7 cells. We found that origins are preferentially positioned at promoters of highly active genes, and that a statistically significant correlation exists between the positioning of origins and the location of H3K4Me3 and Pol-II binding sites on chromatin. Overall, our results suggest a strong link between the distribution of origins of DNA replication and features of the genome related to gene expression and chromatin organization.

## Results

### Overall strategy

To study the global distribution of origins of DNA replication in human chromosomes we have followed a strategy which utilizes a DNA microarray hybridization assay to measure the enrichment of short nascent strand DNA obtained from asynchronous proliferating cells. Briefly, nascent DNA strands released from total genomic DNA by heat denaturation, were size fractionated on a 5–30% sucrose gradient. A selected pool of fractions containing DNA in the 0.7–1.5 kb size range, were subjected to digestion with λ-exonuclease, and the resulting DNA constituted our test DNA. Total genomic DNA, obtained from the same cell line and sonicated to a similar size range constituted our reference DNA. In all our preparations, the test DNA showed at least a 20-fold enrichment of origin relative to adjacent non-origin sequences, as determined by a real time PCR-based nascent DNA abundance assay. In contrast, the same assay performed with the reference DNA yielded a ratio close to 1. Thus the enrichment found with our test DNA fulfilled the criterion of at least 10-fold enrichment for a site to be considered an origin of DNA replication [12]. Both test and reference DNAs were then labeled with Cy-5 and Cy-3 dUTP derivatives, respectively and hybridized to a custom made DNA tiling microarray containing 50–60 nt DNA probes staggered in 50–60 bp steps and spanning a total of 33.5 Mb of human DNA (Supporting Tables S1). Repeat DNA sequences encountered in these regions were masked and excluded in the array. A signal-processing algorithm (see Statistical Methods Supplement) was utilized to analyze the microarray data and to identify peaks indicating the positions on the genome where short nascent DNA strands were enriched. These sites defined the locations of origins of DNA replication.

### Localization of origins of DNA replication in MCF-7 cells

Our initial studies were performed with DNA obtained from the breast cancer cell line MCF-7. Prior to the isolation of short nascent DNA, the exponential growth of the culture was verified by fluorescent activated cell sorter (FACS) analysis (a representative FACS profile for an MCF-7 preparation is shown in Supporting Figure S11, and the quantification of cells in each cell cycle phase for all the preparations used in this study is shown in Supporting Tables S1). The nascent nature of the DNA pool in the 0.7–1.5 kb size range was confirmed by employing a real time PCR-based enrichment assay focused at a previously reported origin of DNA replication around the human ribulose phosphate epimerase (RPE) gene [13]. We found that the enrichment values (a) were maximal at the fractions containing DNA in the short size range used for array experiments (0.7–1.5 kb); (b) they progressively decreased as the size range of the DNA increased; and (c) this activity was not significantly affected by prior treatment of the nascent DNA preparation with either RNase or λ-exonuclease (Supporting Figure S1). In addition, in DNA preparations obtained from estrogen-deprived MCF-7 cell (which by FACS analysis showed an arrest of about 80% at G1), origin enrichment was only found upon progression of MCF-7 cells into the S-phase following estradiol addition (Supporting Figure S1). These observations strongly indicated that the 0.7–1.5 kb pooled DNA contained bona fide short nascent DNA strands arising from actively proliferating cells.

**Figure 1:**
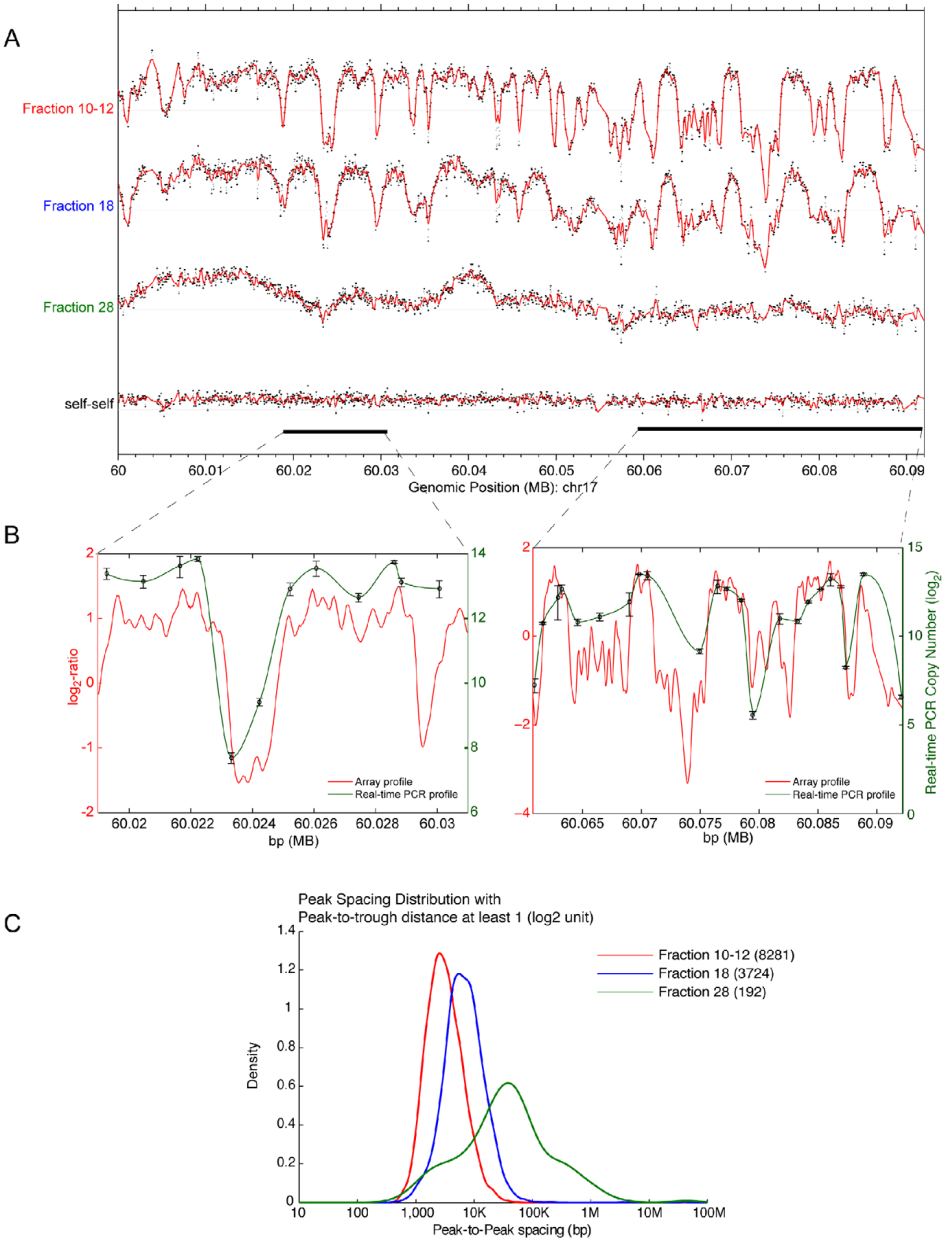
Comparison of nascent DNA enrichment profiles obtained with MCF-7 nascent DNA fractions of increasing size. (A) DNA from sucrose gradient fractions containing nascent DNA with a size range of, 0.7–1.5 kb (fraction 10–12); 1.5–3 Kb (fraction 18); and >3 kb (fraction 28) obtained from the same MCF-7 preparation were hybridized to the tiling array. As a control, sonicated total DNA in the size range of 0.7–1.5 kb was utilized as both test and reference DNA (self-self). After hybridization, the test/reference ratios (black dots) were processed to produce a smoothed line (red line). The peaks represent sites where enrichment of nascent DNA (origins) occurred along a 90 kb region of chromosome 17 (B) Comparison of origin profiles along two (13 kb and 43 kb respectively) selected regions on chr17 (indicated as bars in Figure 1A) obtained from either array data (red line) or from real-time PCR data (black line). (C) Peak spacing densities derived from nascent DNA enrichment profiles for the three DNA size range preparations hybridized to the array. Peaks were detected using a Savitzky-Golay filter with a 500 bp moving window over the genomic regions covered by the array. The number of peaks detected in each of the preparations is indicated in parentheses.

Upon hybridization of the 0.7–1.5 kb nascent DNA (test DNA) and similarly size sheared total DNA (reference DNA) to the tiling array, we observed a strong short-range autocorrelation among neighboring probes (Fraction 10–12, Fig. 1A), which was absent in the input\input hybridization (self-self, Fig. 1A). These results suggested that the peak signals observed with the short DNA arose from the enrichment of regionally localized DNA sequences in our DNA preparation. If the pattern of peaks and troughs derived from short nascent DNA, as opposed to randomly broken DNA fragments, we predicted that the peak signals would diminish in fractions containing larger DNA fragments. To test this, we examined fractions containing nascent DNA in the ranges of 1.5–3 Kb, and ≥3 kb DNA (Fraction 18 and Fraction 28, respectively, Fig. 1A). As predicted, their profiles were both quantitative and qualitatively different from that of the fraction 10–12 DNA pool, yielding progressively fewer and broader peaks. We interpreted these results as indicative of enrichment for origins of DNA replication in the 0.7–1.5 kb fraction, that decreased with fragment size, since the ratios of signals emanating from test versus reference DNA were greatest in the short nascent DNA and declined in fractions with larger fragments.

To validate the accuracy of our origin mapping method we used two approaches: First, we calculated by real-time PCR the copy number at positions of the array showing 13 peaks and 22 troughs on two contiguous regions of chromosome 17 (for the list of primer sets used see Supporting Tables S1). As shown in Figure 1B, our real time PCR results paralleled the patterns observed with the microarray assay, thus validating the test/reference DNA ratios deduced from the array hybridizations. Second, we determined the nascent DNA enrichment at four chromosomal regions, embedded into our DNA array (Supporting Tables S1) that served as internal controls for origins of DNA replication, and positioned around the c-myc, β globin, Lamin B2, and the RPE genes, respectively [13], [14], [15], [16]. We found that nascent DNA peaks detected in the array occurred in proximity or, at each one of these origins, with a mean distance of less than 500 bp between predicted origins and centers of known origin windows (Supporting Tables S1). Finally, short nascent DNAs from three independent MCF-7 preparations produced the same array profile.

**Figure 2:**
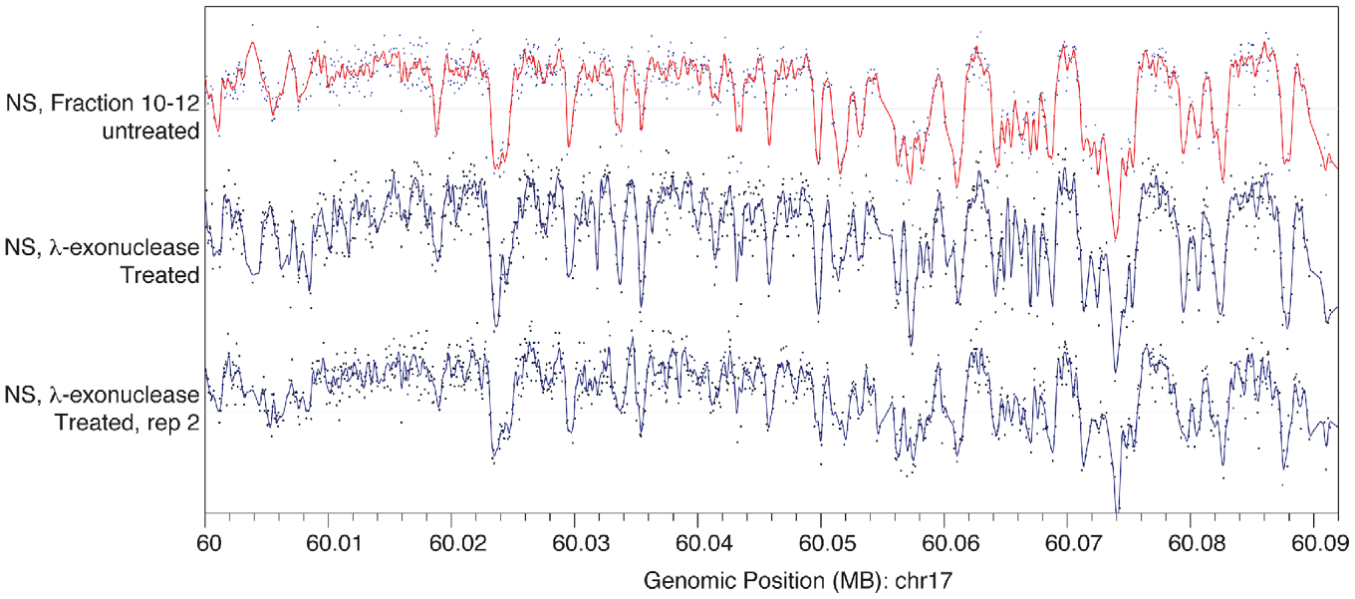
λ-exonuclease treatment of nascent DNA does not alter the array profile. Array hybridizations results with MCF-7 short nascent DNA untreated or treated with λ-exonuclease, prior to hybridization, were compared along a 90 kb region of Chr17. The top panel shows the profile of a λ-exonuclease untreated preparation (NS fraction 10–12). The lower panels are two independently isolated samples (NS71 and NS73) after λ-exonuclease treatment (NS Lambda exo, and NS lambda exo, rep 2 , respectively).

**Figure 3:**
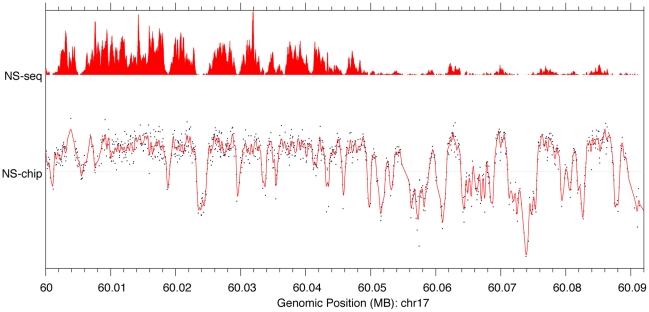
Correlation of nascent DNA enrichment profiles by DNA microarray assay, and DNA sequencing. Average array enrichment results (NS-chip) from two independent MCF-7 preparations were compared to those obtained from high throughput DNA sequencing of MCF-7 short nascent DNA after λ-exonuclease digestion (NS-seq). The region of comparison comprises about 90 kb on Chr17. A comparison over a larger region is presented in Figure S2.

To further ascertain that the array profile obtained did not arise from contaminating short double stranded DNA fragments, we obtained two independent nascent DNA preparations from MCF-7 cells (NS71 and NS73) and treated them with λ-exonuclease, following a standard protocol [17]. Upon hybridization of the λ-exonuclease resistant DNA preparation to the array, we observed that the peak profile obtained with these two preparations was almost indistinguishable from that obtained without λ-exonuclease treatment (Figure 2), indicating that the peak profile observed did not arise from contaminating DNA. Finally, to rule out the possibility that a hybridization bias may be responsible for the peak profile obtained, we determined the abundance of DNA fragments present in our short nascent DNA preparation using high throughput DNA sequencing, and compared that profile to the one found through the array method. To this end, we obtained an independent λ-exonuclease-resistant nascent DNA preparation from MCF-7 cells, in the size range of 400–800 nt, which was then converted to double stranded DNA, using DNA polymerase I Klenow fragment and random primers. This DNA was then sequenced using the Illumina Genome Analyzer II. The average of three independent sequencing reads (named NS-seq) were aligned to the UCSC genome browser hg18 build, then converted to hg16 (liftOver, UCSC) for comparison to DNA microarray data (named NS-chip). Figure 3 illustrates the significant correlation between the position of both NS-seq and NS-chip tracks along a 100 kb region of Chr17. This correlation is not confined to regions of high sequence tag abundance but also extends to less abundant regions (Supporting Figure S2). Altogether, the concordance of the results obtained by these two distinct methods strongly supports the enrichment profiles in nascent DNA observed in our DNA array corresponding to the location of putative origins of DNA replication in MCF-7 cells. It is interesting to note that while there is a good correlation between the positions of peaks observed with both methodologies, a much larger range of peak heights is observed with the sequencing technique (Figure 3). This different peak profile may reflect the higher sensitivity obtained with the sequencing technique.

### Distribution of origins of DNA replication is similar in different cell lines

To investigate if the peak profiles varied between cell lines, we compared the MCF-7 profile to that of BT-474 another breast cancer cell line, as well as, to that obtained with H520, a lung cancer cell line.

We calculated the number of origins detected in all chromosomal regions contained in the array for peaks with a height >1 (log2 units), allowing an overlap in independent replicate experiments of at least 750 bp. In MCF-7, we calculated the number of origins detected with both the short nascent DNA (fractions 10–12) as well as with fractions of increasing size (fractions 18 and 28). The short nascent DNA pool yielded 8281 peaks, and as expected, the number of peaks decreased considerably as the average size of the nascent DNA increased in size (3074 peaks and 192 peaks were found in fractions 18, and 28, respectively; Fig. 1C). Similarly the spacing between peaks or inter-origin distances, were substantially shorter in the fraction 10–12 pool (about 4 kb) compared to about 10 kb in fraction 18, and 1 Mb for fraction 28, respectively (Fig. 1C).

**Figure 4:**
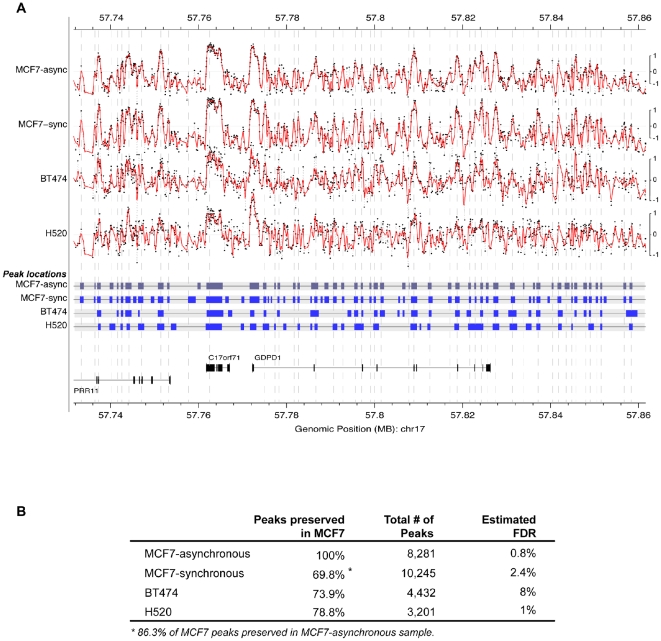
Pattern of origin profiles among different nascent DNA preparations, along a 124 Kb region of chromosome 17. (A) Top panel: origin peaks (peak to through ratio = 1 (log2-ratio)) obtained with both asynchronous (async) and synchronuously (sync, S-phase) growing MCF-7 cells, as well as with asynchronously growing BT-474 and H520 cells. Raw ratios (black dots) and smoothed ratios (red lines) are presented for each preparation. Due to the relatively weak signal observed with both BT-474 and H520, compared to MCF-7, we magnified their corresponding tracks by a factor of 1.5. Center panel: Comparison of the position of putative origin sites among the four different nascent DNA preparations. Origins are represented as gray or blue boxes reflecting the half-peak width (measured at half-peak height) around the highest point of each enrichment peak. Gray vertical lines indicate the position of the highest point of each origin observed with the asynchronous MCF-7 preparation. Bottom panel: Diagrammatic representation of the genes present in the analyzed region of chromosome 17. (B) Statistical comparison on the concordance of origins among the different nascent DNA preparations. Peak concordance between two nascent DNA preparations is calculated by counting overlapping half-width of peaks over the entire region covered by the array. FDR values were calculated as described in the Statistical Methods Supplement.

When we compared the inter-origin distances among BT-474 and H520 cell lines, we found that they fell within the range found in MCF-7 cells (3–5 kb; Supporting Tables S1), a spacing similar to that reported in a human lymphoblastoid cell line [18]. Given that the array profiles and spacing were similar in all cell lines, we wished to determine if the distribution of origins was also similar. To this end, we measured the concordance of origin positions across all the chromosomal regions covered in the array. We found a high level of concordance among the three different cell lines. Figure 4A provides an example of this concordance for a 130 kb region of Chr17 in all the cell lines. Overall, in two independent MCF-7 replicates, the concordance of origins was found to be 86% (Fig. 4B). The comparison with an MCF-7 synchronized sample (see methods section) yielded about 70% concordance. When compared to the other breast cancer cell line BT-474, a 74% concordance with the MCF-7 origins was observed. This high percentage of concordance was also maintained in the lung cancer cell line H520 (79% concordance; Fig. 4B). A false discovery rate (FDR) analysis showing a value of <4% confirmed the statistical significance of our findings (Fig. 4B). These results strongly suggest that the global distribution of origins is largely similar in all three cancer cell lines studied.

### Origins of DNA replication are enriched at the 5′ ends of expressed genes

**Figure 5:**
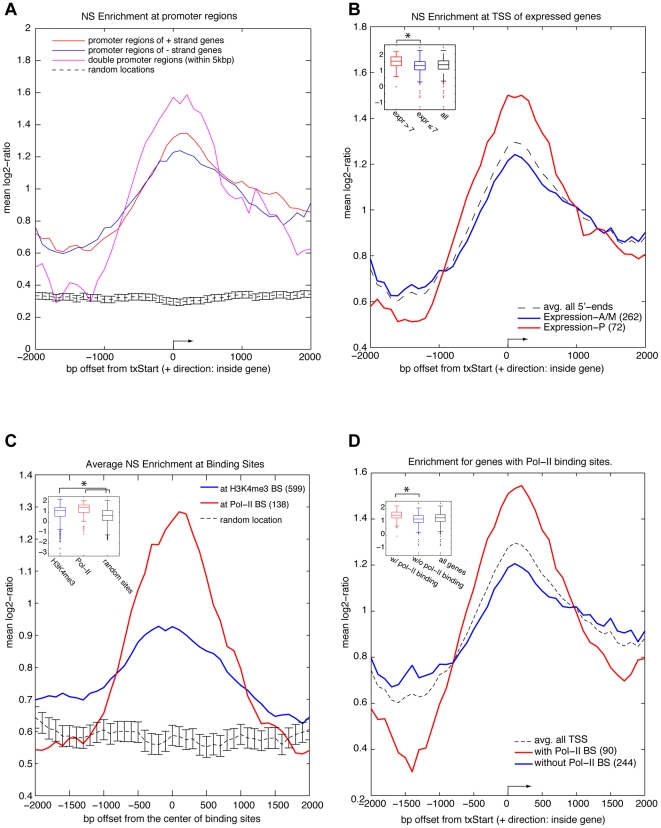
Association of origin peaks with transcription initiation in MCF-7 cells. (A) Association of origin peaks with promoter regions. Composite origin profile centered at the 5′-ends of all genes present in array. The start site and direction of transcription of an average gene are indicated by an arrow. Black lines with standard error bar denote the position of 2000 sites randomly selected to develop a reference line. (B) Association of origin peaks with promoter regions of actively expressed genes. Composite origin peak profiles around the 5′ ends of genes present in the array whose log-2 expression level was greater than 7 (Expression-P, N = 72) or less than 7 (Expression-AM, N = 262). The start site and direction of transcription of an average gene are indicated by an arrow. Inset indicates the statistical significance (t-test, p = 5.8×10^−6^) between the origin peak profile at TSS on expressed genes versus less-expressed genes. (C) Association of origin peaks with chromatin containing H3K4me3 or RNA Pol-II binding sites. Composite origin peak profile around the center of either H3K4me3 (N = 599) or Pol-II (N = 138) binding sites present in the array. Black lines with standard error bar denote the position of 599 sites randomly selected to develop a reference line. Inset indicates the statistical significance (t-test, p<10^−10^) between the initiation profile at the center of the binding sites versus at random sites. (D) Association of origin peaks with genes showing Pol-II binding sites. Composite nascent DNA peak profile around the center of the transcription start site, in genes with (N = 90), or without (N = 244) Pol-II binding sites. The start site and direction of transcription of the average gene are indicated by an arrow. Inset indicates the statistical significance (t-test, p = 3×10^−6^) between the origin peak profile at TSSs on genes with or without Pol-II binding. Details of composite profile generation are described in the Statistical Methods Supplement.

To examine the relationship of replication initiation with transcription, we initially compared the location of known transcription start sites (TSS) contained in our array to the pattern of origin peaks obtained in MCF-7. Using a window of 500 bp to define the positioning of peak signals, a composite origin profile at the 5′-end of all genes present in the array was generated. We observed a significant enrichment near the transcription start sites of genes covered by the array (Fig. 5A). This enrichment was even more evident for adjacent genes transcribed in opposite directions. To assess the statistical significance of these findings we analyzed 2000 positions selected at random within the genomic regions covered by the array. A t-test comparison at TSS for the random sample and the origin peaks demonstrated a highly significant difference (p<10^−41^, Fig. S3). No enrichment was found at the 3′ end of genes (Supporting Figure S4). However, the TSS enrichment was also observed with synchronized MCF-7, and with the BT-474 and H520 cell lines (Supporting Figure S5). Next, we investigated the relationship of replication initiation to gene expression level. Using an Affymetrix data set [19] and a cut off of seven units (log2), for highly transcribed genes, we found that origin peaks at TSS were significantly more enriched in highly expressed genes compared to low/unexpressed genes (Fig. 5B; t-test, p = 5.8×10^−6^). It is important to note that the genome coverage of our array is distributed almost evenly among genic and non-genic regions (Supporting Figure S6), therefore the observed enrichment of origins at promoters sites does not derive from a gene dense array design. Our results are also consistent with recent reports which point to the association of human and mouse origins with transcriptional initiation [20], [21], [22].

### Origins of DNA replication correlate with the positioning of non-genic conserved DNA elements

**Figure 6:**
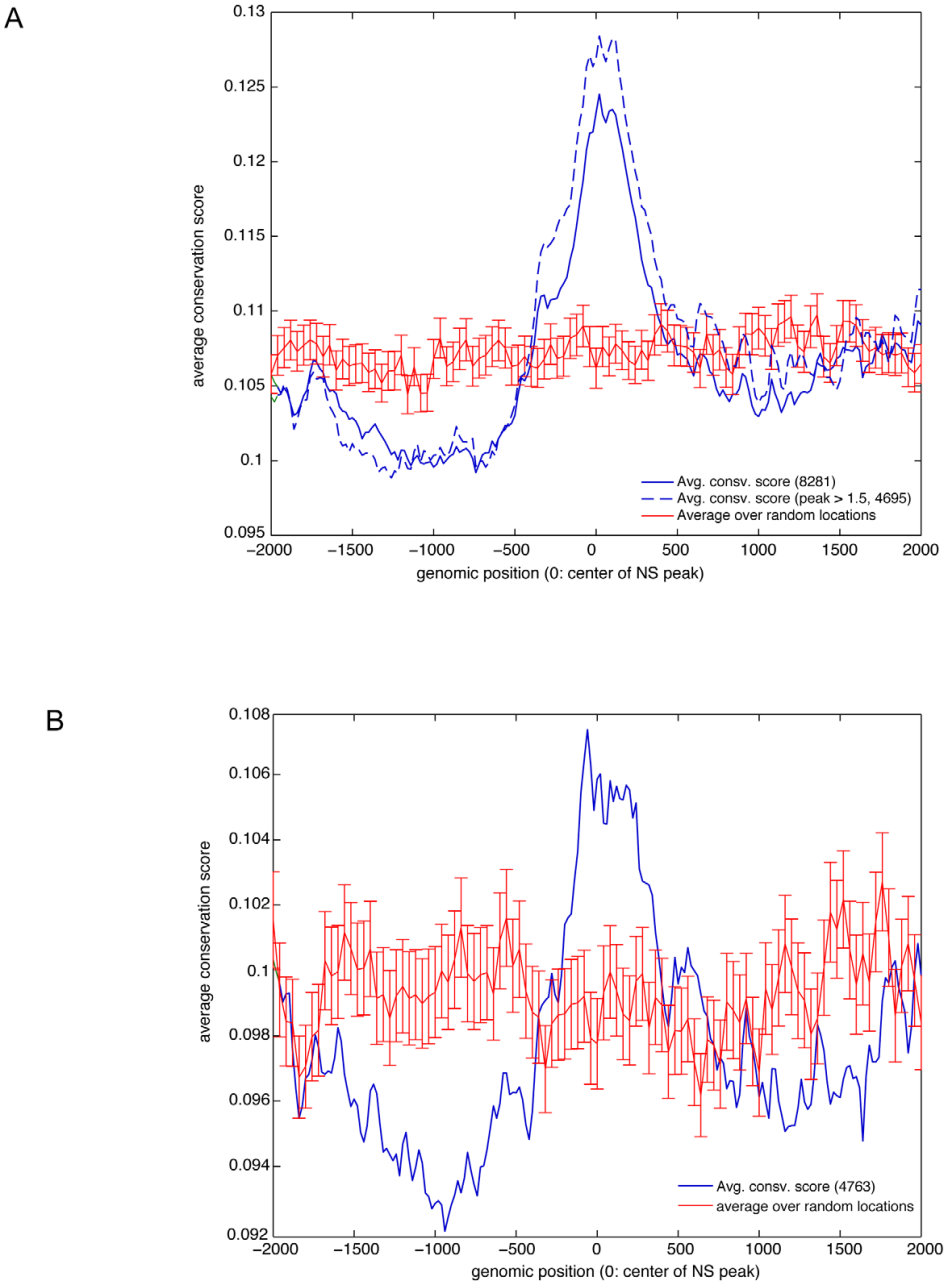
Association of origin peaks with evolutionarily conserved DNA sequences. (A) Composite average conservation score around the highest point of origin peaks (peak to trough ratio >1 log-2 scale; total of 8281 initiation sites; or peak to trough ratio >1,5 ; total 4695 sites). Red lines with standard error bar denote the position of an equivalent number of sites randomly selected to develop a reference line. The statistical significance between the conservation score at the center of nascent DNA peaks with peak to through ratio >1 was highly significant (t-test, p = 1.1×10^−14^) (B) Composite average conservation score of non-genic conservation sequences around the highest point of origin peaks (peak to through ratio >1 log-2 scale; total of 4763). Red lines with standard error bar denote the position of an equivalent number of sites randomly selected to develop a reference line. The statistical significance between the conservation score of non-genic conserved sequences at the center of origin peaks with peak to trough ratio >1 was found to be statistically significant (t-test: p = 2.9×10^−4^).

Because origin peaks were not confined to genes or their 5′ends, we sought to determine if other features of the genome were significantly related to their localization in intergenic regions. DNA sequence comparison of the human genome with other vertebrates has uncovered significant conservation of non-coding DNA sequences suggesting a functional role for these sequences [22]. Visual inspection of the conserved sequences among the human, chimpanzee, mouse, rat, and chicken genomes (UCSC genome browser hg16 build, table mxPt1 Mm3RnGg_pHMM) along the regions covered by our array suggested a correlation of origin peaks with the position of conserved elements. We therefore developed a composite average conservation score around the highest point of the origin peaks (peak heights with at least 1 or 1.5 log2-fold changes). Fig. 6A (green lines) demonstrates an association between the average conservation score with the highest peak enrichment point (solid and dashed green lines for peak/trough ratios of >1.0, and >1.5 log-2 fold, respectively). At peak height log-fold >1.0, the Pearson correlation coefficient was found to be 0.9524, p = 1.19×10^−30^. To further assess the significance of this finding, we selected a similar number of locations at random and calculated the average conservation scores along these locations (Fig. 6A, red line). No significant correlation was found. In contrast, a t-test performed to compare the average conservation score at origin peaks versus random locations was found to be highly significant (p = 1.1×10^−14^). To ascertain if the correlation between origin peaks and conserved sequences also held for non-genic regions, we selected for analysis intergenic regions that were separated by at least 1000 bp from the nearest genes on either end of the gene free segment (an illustration of such region is shown in Supporting Figure S12). Randomly selected sites were subjected to the same criteria. The results shown in Fig. 6B indicate that a highly significant correlation still remains (Pearson correlation coefficient = 0.915, p = 9×10^−24^) at these conserved non-genic regions. When compared to the randomly selected sites the t-test p-value (p = 2.95×10^−4^) was also found significant. Similar results were found in the other cell lines used in this study (Supporting Figure S7). An example of the association of origins with evolutionarily conserved regions is illustrated for a 50 kb intergenic segment on chr17 containing several highly conserved sequences (Supporting Figure S12). These results are consistent with the possibility that evolutionarily conserved elements define functionally active chromatin available as preferred sites of replication initiation.

### Chromatin binding sites for H3K4me3 and PolII correlate with the position of origins of DNA replication in MCF-7 cells

To further evaluate the presence of origin enrichment in regions of active promoters in MCF-7 cells, we determined by chromatin immunoprecipitation (ChIP), the positions of H3K4me3 and Pol-II chromatin binding on our array (ChIP on chip). Consistent with previous reports [23], [24], we found enrichment of H3K4me3 and Pol-II binding at sites of transcription initiation (Supporting Figure S8). Within 1 kb from the TSS, about 52% of all annotated promoters in our array were found to be enriched for H3K4me3, and 27% were found to be occupied by Pol-II. Interestingly, a composite origin profile around the center of either H3K4me3 (599 sites) or Pol-II (138 sites) binding sites revealed a strong correlation (Fig. 4C). A t-test of this association versus a sample extracted from the array containing 599 sites chosen at random showed a significant difference (Fig. 5C; p<10^−10^). We also compared the association of origins with genes harboring (N = 90) or lacking (N = 244) Pol-II binding sites. Fig. 5D shows that a stronger origin association is found at TSSs of genes harboring Pol-II binding sites (t-test, p = 3×10^−6^). These results clearly suggest that the open chromatin structure at these sites may drive the positioning of proteins involved in the initiation of DNA replication. Remarkably, in every nascent DNA preparation tested, origins with strong enrichment were consistently positioned at sites concordant with both Pol-II and H3K4me3 binding (Supporting Figure S9).

## Discussion

In the present study through the application of high resolution DNA array and high throughput DNA sequencing technologies, we have considerably expanded the range and sensitivity of a nascent DNA enrichment assay used to determine the position of putative origins for DNA replication in about 1% of the human genome. We have found that the apparent distance between putative origins is about 3–5 kb in all cancer cells lines tested, a much shorter distance than that deduced from single molecule studies of DNA replication [25], [26]. If all these origins were active in a single cell the genome would complete its duplication in a fraction of the duration of the S phase. These results strongly support the current notion, largely based on studies in the budding yeast and embryonic systems, that eukaryotic genomes contain more initiation sites than those required to complete replication, not all of which are used in each cell cycle [7], [10], [11], [27]. In this context, it should be pointed out that since the results obtained by both DNA microarray and DNA sequencing technologies only provide an average profile of origin activation in a population of cells, these data to not define the origin distribution profile in individual cells. Therefore it is likely that while origin spacing appears to be short when averaged across the population, this most likely reflects a stochastic pattern of origin activation at larger intervals in individual cells rather than unique pattern shared in all cells. The pattern of peaks and troughs that we observe in nascent strands could therefore be regarded as defining the local probability of a replication initiation event [28]. To gain a better understanding about origin activation in human cells and how cell lineage or environmental changes disturb replication profiles, it might be necessary to complement genome–wide population studies with single cell analysis. Indeed in one such study in the budding yeast where DNA combing combined with DNA fiber fluorography was used to deduce the replication profile in Chr VI it was found that all yeast VI chromosomes showed different replication profiles when analyzed as single molecules, while recapitulating microarray data when averaged [29]. It would also be of considerable interest to compare origin profiles between isogenic normal and cancer cells to determine if origin selection is altered in transformed cells.

Our findings offer a glimpse of the relationship between DNA replication and other aspects of mammalian chromosome function by clearly establishing that origins of replication are non-randomly distributed with respect to genome landmarks. These include the transcription start sites of active genes and conserved elements in intergenic regions. These results may be the consequence of easier access by the DNA replication machinery to specific regions. Transcription start sites must contain relatively open chromatin and are frequently marked by nearby nucleosome free DNase hypersensitive sites [30]. Thus the formation of a replication initiation complex may be favored at these locations. Our findings confirm and extend recent results found with mouse ES cells [31] and human cells [32] regarding mammalian replication origins and their proximity to transcription start sites, as well as, to RNA Pol-II, and histone H3K4Me3 chromatin binding sites. A recent report [21] suggests that DNA over-replication of short DNA fragments around promoter regions may also account for the apparent enrichment of origins around transcription start sites. While it is not clear whether the short fragments observed in the study of Gomez and Antequera [21] elongate into mature replicons, our data is consistent with this novel and provocative finding, and it is possible that our nascent DNA preparation may also contain some of these short over-replicated DNAs. However, given that the genome coverage in our array is almost equally partitioned among genic and non-genic regions, and the fact that we do not observe a strong bias for the localization of origins in the genic regions, other factors must determine the placement of origins in non-genic regions. The function of intergenic conserved elements is largely unknown [33]. Some may function as enhancers for distant genes and might therefore also be accessible to the formation of nucleoprotein complexes. Our results strongly suggest that many of these evolutionarily conserved elements are indeed functionally active in at least one critical process, initiation of DNA replication. We have found that active origins in intergenic regions are strongly associated with conserved sequences. However, this association is not completely explained by H3K4Me3 modification around these sites since nucleosomes containing this epigenetic marker are only slightly enriched at conserved sequences in the intergenic regions represented on our array (Supporting Figure S10). These results suggest that the increased probability of replication initiation at conserved intergenic sites must be determined by another as yet undescribed property of these regions.

Further whole genome investigation, coupled with studies on individual DNA molecules, will be useful to identify DNA elements and their associated chromatin features including, epigenetic modifications, participation in higher order structures, and function in regulating gene expression which enhance the likelihood of forming an active replication initiation complex. This information should provide us with a deeper understanding of the process of replication origin selection in mammalian cells.

## Materials and Methods

### (a) Cell lines and FACS analysis

Breast cancer cell lines MCF-7 and BT-474, and lung cancer cell line H520 were obtained from the American Type Culture Collection (Manassas, VA). Cells were grown according to recommended specifications, to about 70% confluence. An aliquot of the cell culture, corresponding to about 10^6^cells was set aside for Fluorescent Activated Cell Sorting (FACS) analysis. The aliquot of cells was prepared for FACS analysis using the cellular DNA flow cytometric analysis kit (Roche, IN) following the manufacturer's specifications. The percentage of cells in the S phase served as a good predictor of the amount of nascent strand DNA available in the preparation (see Supporting Tables S1).

### (b) Isolation of short nascent strand DNA

The procedure previously employed to isolate nascent DNA (14) was followed with minor modifications. About 2–5×10^8^cells were washed in PBS and collected by centrifugation. Cells were lysed with SDS in presence of Proteinase K. DNA was extracted with phenol and chloroform, precipitated by centrifugation with ethanol in 0.3 M sodium acetate, and resuspended in TE (10 mM Tris-HCl, pH 8.0; 1 mM EDTA) buffer. The re-suspended DNA was denatured by incubation in boiling water for 12 min, quenched in ice for 6 min, and applied onto a linear 5–30% neutral sucrose gradient. After centrifugation of the gradient in a Beckman SW28 rotor for 24,000 rpm for 20 hrs at 15°C, the gradient was fractionated using an ISCO 185 fractionator. One ml fractions were collected and the linearity of the gradient assessed by measuring the refractive index of every third fraction. Gradients were highly reproducible with regression line R^2^ values larger than 0.99. The reproducibility of the gradients allowed us to identify fractions corresponding to the desired DNA size range, which in our experience falls around a refractive index of 1.35. About 80 µl of every gradient fraction was concentrated 10-fold and analyzed by gel electrophoresis in 1% agarose to confirm the DNA size range in the fractions. Fractions containing DNA in the range of 0.7–1.5 kb in length were pooled and dialyzed against TE buffer. Two independent MCF-7 0.7–1.5 kb DNA pools (NS71 and NS73) were treated with λ- exonuclease following a standard protocol [17]. For comparison purposes an equal amount of total sheared (0.5–1.5 kb) was also digested under the same conditions. The quality and abundance of short nascent strands in the DNA preparations was assessed by real time PCR (Supporting Figure S2).

### (c) DNA array design and hybridization

60-nt probes spaced by an average length of 50–60 nt were designed to cover about 34 Mb of human DNA and distributed in several chromosomal regions (Supporting Tables S1). A 5 Mb region of Chr20q12.13 was represented by DNA probes to both strands of this DNA region. This served as an internal control region to assess the reproducibility of signals emanating from the same DNA region. Finally, we eliminated potentially cross-hybridizing DNA probes by checking the uniqueness of each probe in the human genome. The hybridization protocol followed was essentially similar to one used for comparative genomic hybridization (CGH) to NimbleGen arrays (NimbleGen Systems Inc.). The test DNA consisted of selected DNA fractions from the sucrose gradient with or without λ-exonuclease treatment. The reference DNA sample, corresponded to DNA obtained from the same cell line from which the nascent DNA preparation was originated. This DNA was sheared by sonication to yield an equivalent range in size fragments as the test DNA. Each of the DNA samples was independently labeled by random priming with dye-modified dUTPs (e.g. Cy5-or Cy3-), and then combined before hybridization to a NimbleGen array using a MAUI hybridization system at 42°C for 16–20 hrs. The slide containing the array was then removed from the MAUI hybridization chamber while immersed in wash buffer I (1X SSC, 0.05% SDS), placed in a slide rack containing wash buffer I and washed twice in the same buffer for 5 min with agitation. The slide was transferred to wash buffer II (0.1X SSC) and the washing repeated as before. The slide was then removed from the slide rack and dried by centrifugation (1500 rpm for 3 min) prior to scanning.

### (d) Array Data Analysis: Feature Extraction

The hybridized microarray was scanned with the Agilent Microarray Scanner (Agilent Technologies, Santa Clara, CA). Two color images were analyzed by NimbleScan software (v2.1, NimbleGen, Madison, WI) and exported with probe intensities from both channels. The data were subsequently converted, without normalization, to log2-ratios in SGR and BED formats, for data visualization in the Affymetrix Integrated Genome Browser (IGB, www.affymetrix.com) or as custom tracks in the UCSC genome browser.

### Data Analysis

(See Statistical Methods Supplement for additional details.) A peak finder algorithm was developed as follows. Briefly, we first ordered the data according to genomic location, re-sampled the data (log2-ratio) to achieve equal 50 bp spacing and then interpolated to 25 bp spacing in order to meet the requirements of subsequent methods. We then used the Savitzky-Golay convolution smoothing kernel [34] to smooth the data to the degree needed (span  = 7 was the default choice). Peaks were then detected with the first derivative. We determined the minimal detectable peak height by using the error derived from smoothing filtering. We ignored peaks with height less than the minimum detectable peak. After the peak-finder algorithm had identified all of the peaks, the peak height density plot was generated. Self-self hybridization peak heights were mostly less than 1.0 (log2-ratio). By setting a peak height threshold at 1.0, the peak spacing density, which reflects the peak-to-peak distance, was generated by counting only peaks higher than 1.0.

### (e) Real Time PCR

Real time PCR analysis was performed on all DNA preparations to ascertain their enrichment for short nascent strands. In addition to origin/non origin sites previously characterized by others around the lamin B2 gene and the β-globin locus, two bona fide sites, around the ribulose phosphate epimerase gene (14), an origin site, STS36.8, at position 211060206211060430 on Chr2q34, and a non-origin site, STS98.4, at position 211121797–211122038, on Chr2q34, were used as markers to determine the enrichment for initiation sites in the fractions containing our short nascent DNA pools [13]. For our real-time PCR assays, fractions around the DNA size range of 0.7–1.5 kb were brought to a concentration of about 10 ng/µl, and 2 µl of these preparations were used for real time PCR assays. As a reference marker, fraction number 25 corresponding to the lower third of the sucrose gradient, was also analyzed. PCR reactions were carried out as previously described [13]. For each primer set used, MCF-7 total DNA which had been sheared by sonication to a size range of 0.5–1.5 kb was diluted to give 20,000, 4,000, 800, 160, and 32 genomic copies per µl respectively. 2 µl of these dilutions were run in triplicate as copy number standards. As a negative control 2 µl triplicate aliquots of water were used. Copy numbers for STS36.8 or STS98.4 were estimated from a standard curve obtained with the samples containing known amounts of genomic equivalents. Ratios of the copy number at STS36.8/STS98.4 larger than 10, were indicative of a good nascent DNA strand preparation. As an internal control, the ratio of STS36.8/STS98.4 in fraction number 25 was always found to yield a value close to 1. Fractions containing the highest ratios of STS36.8/STS98.4 were then pooled and used for hybridization to the DNA tiling microarrays. Once initiation sites along each one of the chromosomal regions represented in the DNA tiling microarray were identified, we also used real-time PCR to validate both initiation and non-initiation regions by selecting STS/primer sets from peak regions and adjacent troughs, and their abundance in short nascent DNA strand preparations determined (for a list of primer sets used see Supporting Tables S1).

### (f) Cell synchronization

Cells were grown in appropriate media until they reach 60% confluence. At this point the cells were placed in a charcoal-treated media and kept in this media for 48 hrs. Estradiol (10 nmoles/ml) was added to the media and at times 0, 2, 4, 8, 12, and 18 hrs after the addition of estradiol, aliquots containing about 10^6^cells were taken and processed for FACS analysis as described above. Once we obtained synchronization of the cells as demonstrated by FACS analysis, nascent strand preparations from each one of the time points were prepared as described above, and the copy number at STS36.8 and STS98.4 on chromosome 2q34 was determined by real time PCR as indicated above. As expected in the time points preceding the shift of the cell culture from G1/G0 to the S phase of the cell cycle, the majority of the cells had been arrested in G1/G0. Accordingly, our real time PCR assays at these time points yielded an STS36.8/STS98.4 copy number ratio that approximated to one, indicating that short nascent DNA strands, corresponding to activated initiation sites, have not yet been produced. As the cells entered into the S phase, the proportion of cells leaving the G1/G0 phase were assessed both by FACS analysis and real time PCR assays. Cultures showing a maximal entry into S phase, around 12–14 hrs after estradiol addition (about 2–4 hrs into the S phase), were selected for analysis.

### (g) Chromatin immunoprecipitation

(ChIP) was carried out according to standard protocols using a ChIP-IT kit from Active Motif (Carlsbad, CA), and following the manufacture's instructions with minor modifications. Briefly, MCF-7 cells were crosslinked with 1% formaldehyde at room temperature for 15 minutes. Then the cells were sheared with a VirSonic 100 sonicator for 10 cycles of ten 1-second pulses. After centrifugation, the chromatin contained in the supernatant was collected. Part of it was set aside and served as the input fraction. The rest was immunoprecipitated overnight at 4°C. The antibodies used were: anti-polymerase II antibody (Upstate 05–623) and anti-trimethylated histone H3K4 (Abcam Ab8580). After reversal of crosslinks at 65°C overnight, the ChIP DNA was purified using spin columns provided by the kit. For ChIP-chip, the ChIP DNA was amplified using a ligation mediated-PCR method, as previously described [35]. A second round amplification of 15 cycles was added to increase the yield of DNA. 3 µg of amplified ChIP DNA and Input DNA was labeled with Cy5– dUTP and Cy3-dUTP, respectively, with a BioPrime DNA Labeling System (Invitrogen). The labeled ChIP DNA and Input DNA were then combined and hybridized to the NimbleGen arrays.

### (h) High-throughput DNA sequencing

The procedure described above for the isolation of short nascent DNA was utilized on a culture of exponentially growing MCF-7 cells. After λ-exonuclease treatment of the DNA pool in the size range of 400–800 bp, we synthesized a double stranded DNA population required for massively parallel sequencing using the Klenow fragment of DNA polymerase I (Invitrogen, Carlsbed, CA) and random primers (Invitrogen, Carlsbad, CA). Random priming and DNA synthesis were performed according to the manufacturer's protocol except that the samples were incubated for an hour at 37°C. To insure that the resulting population represented bona fide nascent strands and that random priming did not introduce a quantitative bias, real time quantitative PCR was performed before and after second strand synthesis using origin-proximal and origin-distal primers from two regions that contain known replication initiation sites, the human beta globin and lamin B2 loci, as previously described. This DNA was then submitted for DNA sequencing using the Illumina Genome Analyzer II (Illumina, San Diego, CA). Three independent sequencing reads were merged into one single tag-count list, after aligning to hg18 and filtering of multiple occurrences of identical reads. The alignment results were subsequently down-lifted to hg16 (liftOver, UCSC) to compare to other tracks generated from the tiling microarray technology. Before the comparison, counts data from sequencing were further subsampled into 10 bp spacing, and them smoothed with kernel density function with window size of 500 bp with 50 bp interval (similar to the spacing of the tiling microarray, termed as NS-chip). For comparison purposes, we displayed every track in bar charts and omitted ratios less than 1 (log-ratio less than 0) in NS-chip results.

### (i) Data deposition

The data discussed in this publication have been deposited in National Center for Biotechnology Information's Gene Expression Omnibus (GEO, ttp://www.ncbi.nlm.nih.gov/geo/) and are accessible through GEO Series accession number GSE10917.

## Supporting Information

Figure S1 **Nascent DNA enrichment at a known initiation site for DNA replication, as determined by real time PCR.** (A) Enrichment is maximal in fractions containing DNA in the 0.7–1.5 kb size range. Fractions of the sucrose gradient (from a total of 35 fractions), containing increasing DNA size fragments obtained from MCF-7 cells were analyzed by real time PCR. DNA copy numbers at both STS36.8 (initiation site) and STS98.4 (non-initiation site) were obtained and the ratio of these copy numbers represented the enrichment at the initiation site. Approximate size of DNA fragments: Fr.9 <0.7 kb; Fr.10–12∼0.7–1.5 kb; Fr.15 >1.5–3 kb; Fr.28 >3 kb. (B) Nascent DNA enrichment is not affected by prior treatment of the DNA with λ exonuclease. A pool of fractions from the sucrose gradient containing DNA in the size range of 0.7–1.5 kb obtained from MCF-7 cells was analyzed by real time PCR (MCF-7 NS20) prior or after (Exo) treatment with λ exonuclease. DNA copy numbers at both STS36.8 (initiation site) and STS98.4 (non-initiation site) were obtained and the ratio of these copy numbers represented the enrichment at initiation site. As a control, total MCF-7 DNA in the same size range was analyzed in parallel. Upon treatment with λ-exonuclease the enrichment factor remained the same (around 30 for the nascent DNA, and around 1 for the total DNA). (C) Nascent DNA enrichment at a known initiation site for DNA replication is maximal in synchronized MCF-7 cells entering into the S phase of the cell cycle. MCF-7 cells were arrested in the G1 phase of the cell cycle by keeping the cells for 48 hrs in estrogen-depleted medium. Upon transfer to a medium containing 10 nM estradiol aliquots were taken at times 0, 4, 8, 12, 14, and 17 hrs after estradiol addition. Aliquots were prepared for FACS and nascent DNA analysis. FACS analysis showed that cells entered the S-phase only after 10 hrs of estradiol addition (data not shown). Fraction#11 from the sucrose gradient containing DNA in the size range of 0.7–1.5 kb from each one of the aliquots was independently analyzed by real time PCR. DNA copy numbers at both STS36.8 (initiation site) and STS98.4 (non-initiation site) were obtained, and the ratio of these copy numbers represented the enrichment at the known initiation site.
(TIFF)

Figure S2 **Correlation of NS-seq and NS-chip profiles at a low sequence tag region.** Comparison of NS-seq data with two independent NS-chip data obtained from MCF-7 preparations along a chromosomal region containing a relatively low sequence tag abundance.
(TIFF)

Figure S3 **Enrichment of origin peaks at transcription start sites (TSSs) in MCF-7.** A total of 334 TSSs on single promoters, and 21 TSSs in regions containing two diverging promoters were analyzed for origin peak enrichment using a smoothing window size of 500 bp. The left panel shows a composite plot for all genes centered at the TSS site. The right panel shows the enrichment profiles for genes transcribed in either orientation and for regions containing double promoters. Notice that the origin enrichment is more noticeable in this latter class.
(TIFF)

Figure S4 **Origin peaks are not enriched at the 3**′ **ends of genes in MCF-7.** A total of 316 unique txEnd sites, and 29 unique 3′–3′ sites (diverging transcripts within 5000 bp) present in the array were analyzed for origin peak enrichment using a smoothing window size of 500 bp. The left panel shows a composite plot for all genes centered around the txEnd site. The right panel shows the enrichment profiles for genes transcribed in either orientation and for regions containing double promoters.
(TIFF)

Figure S5 **Enrichment of origin peaks at transcription start sites in all cell lines.** Panels (a–c) show the enrichment of origin peaks at TSSs for short (0.7–1.5 kb) nascent DNA obtained from MCF-7, BT-474, and H520 cell lines , respectively. In each panel the enrichment at both single and regions containing two diverging promoters is indicated. Panels (d–e) show enrichment profiles for longer MCF-7 nascent DNA (1.5–3 kb, and >3 Kb, respectively). Panel f. Enrichment analysis on total sheared (0.5–1.5 kb) self- hybridized MCF-7 DNA is shown as a negative control.
(TIFF)

Figure S6 **Summary of distribution of origin peaks among genic and intergenic regions and their association with promoters and conserved elements for all cell line used in the study** (see Statistical Methods Supplement for detailed description of methods). Distribution of origins in asynchronous cultures of MCF-7, BT474, and H520 cancer lines, among both genic and intergenic regions (representing 43.5%, and 56.5% of all the sequences comprising the array, respectively). Genic regions have been further subdivided among those containing single promoters (dark blue pie segment), and those containing two closely space divergent promoters (light blue pie segment). The number of origins overlapping with evolutionarily conserved sequences in the intergenic region, is also indicated.
(TIFF)

Figure S7 **Association of origin enrichment with evolutionarily conservation scores in all cell lines.** Panels (a–b) show the association of origin peaks with evolutionarily conservation scores for short (0.7–1.5 kb) nascent DNA obtained from BT-474, and H520 cell lines, respectively. Panels (c–d) show enrichment profiles for longer MCF-7 nascent DNA (fraction 18; 1.5–3 kb, and fraction 28; >3 Kb, respectively).
(TIFF)

Figure S8 Association of origin enrichment with (a) Pol-II (blue line), and (b) H3K4Me3 (blue line) chromatin-binding sites in MCF-7, versus random locations (red line). Box-plot of enrichment level is shown in the bottom panels.
(TIFF)

Figure S9 **Correlation between efficiency of origin firing with Pol-II and H3K4Me3 binding along a 300** **kb region of chr17.** The distribution of origin peaks (peaks above the baseline indicated by a horizontal gray bar) obtained with MCF7, BT474, and H520 asynchronous cultures, along a 300 kb region of Chr17 (Chr17∶59,030,000–59,330,000) is shown. The position of RNA Pol-II and H3K4Me3 chromatin binding sites along this region as shown by ChIP on chip analysis is indicated by blue boxes. At the bottom of the figure, the position of three genes (APPBP2, PPMID, and BCAS3) present in this region is also illustrated. The vertical gray lines indicate the correlation between high origin peaks with the position of both Pol-II and H3K4Me3 binding sites.
(TIFF)

Figure S10 **Conservation score enrichment at H3K4Me3 chromatin-binding sites.** Top panel: Average conservation score at intergenic regions, around the center of H3K4Me3 binding site peak. Bottom panel: Conservation score for the entire region covered by the array. The random locations (equal to the number of corresponding binding sites) were sampled from the genome regions covered by the array, and the mean and standard error of log-ratios was plotted (red-colored lines) with error bars at 100 bp intervals.
(TIFF)

Figure S11 **Profile of an exponential asynchronous culture of MCF-7 cells after fluorescent activated cell sorter (FACS) analysis.** The distribution (percentage) of cells among the three major phases of the cell cycle (G1, S, and G2/M) is indicated for both Watson Pragmatic, and Dean/Jett/Fox methods of analysis.
(TIFF)

Figure S12 **Diagrammatic representation of a 300** **kb region of Chr17 containing non-genic evolutionary conserved elements.** The top panel illustrates a 150 kb non-genic region flanked by the 3′-end of the YPEL2 gene and the 5′-end of the DHX40 gene. Bottom panel shows the location of DNA elements showing high conservation score among human, chimp, mouse, rat and chicken (indicated by arrows), within a 50 kb subregion (Chr17∶58,000,000–58,050,000).
(TIFF)

Tables S1 (PDF)
