# Supplementary figures and images for: Preferential Localization of Human Origins of DNA Replication at the 5′-Ends of Expressed Genes and at Evolutionarily Conserved DNA Sequences
Source: PLoS One. 2011 May 13;6(5):e17308. doi: 10.1371/journal.pone.0017308 (PMC3094316; doi:10.1371/journal.pone.0017308)

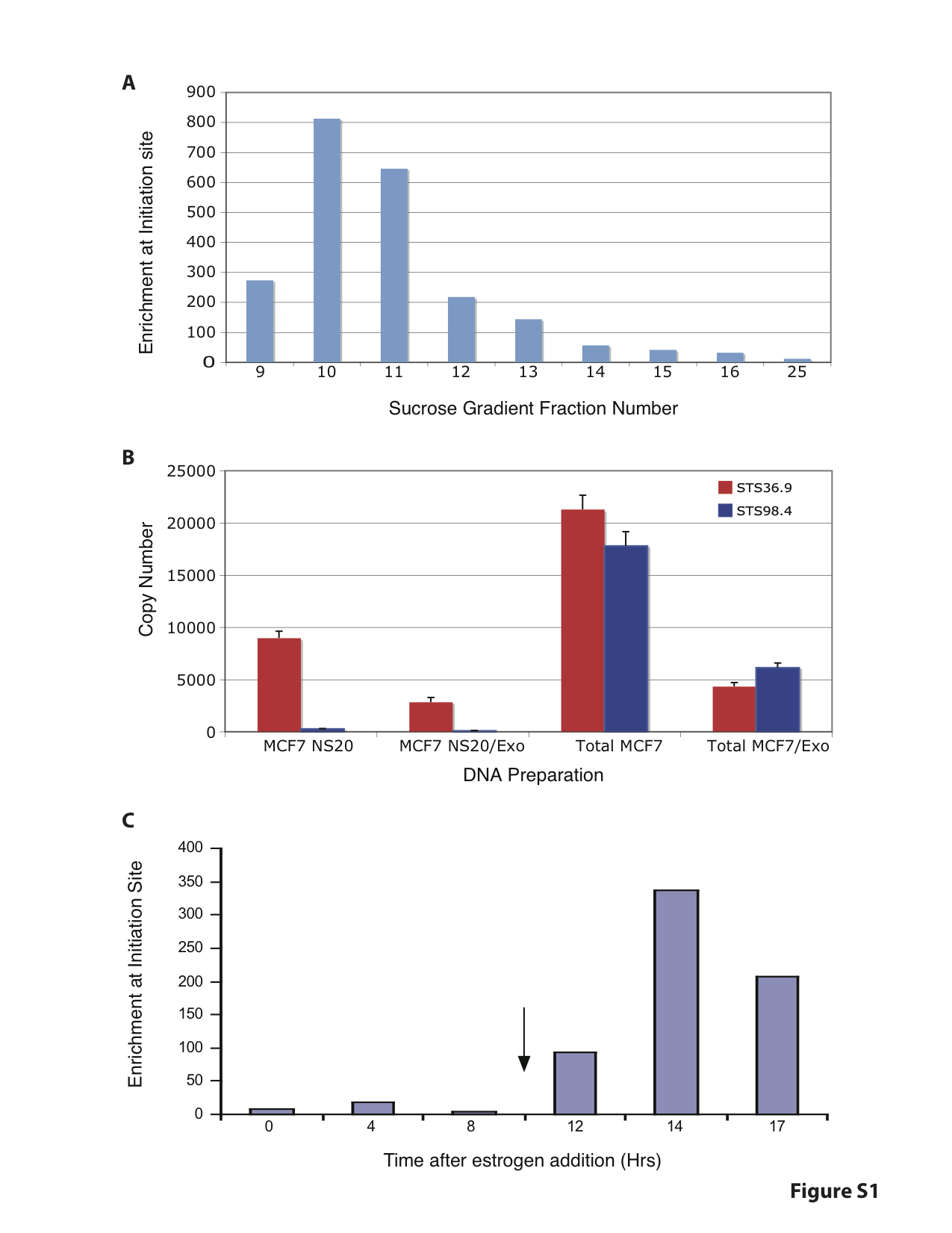

Supplement: Figure S1 — Nascent DNA enrichment at a known initiation site for DNA replication, as determined by real time PCR. (A) Enrichment is maximal in fractions containing DNA in the 0.7–1.5 kb size range. Fractions of the sucrose gradient (from a total of 35 fractions), containing increasing DNA size fragments obtained from MCF-7 cells were analyzed by real time PCR. DNA copy numbers at both STS36.8 (initiation site) and STS98.4 (non-initiation site) were obtained and the ratio of these copy numbers represented the enrichment at the initiation site. Approximate size of DNA fragments: Fr.9 <0.7 kb; Fr.10–12∼0.7–1.5 kb; Fr.15 >1.5–3 kb; Fr.28 >3 kb. (B) Nascent DNA enrichment is not affected by prior treatment of the DNA with λ exonuclease. A pool of fractions from the sucrose gradient containing DNA in the size range of 0.7–1.5 kb obtained from MCF-7 cells was analyzed by real time PCR (MCF-7 NS20) prior or after (Exo) treatment with λ exonuclease. DNA copy numbers at both STS36.8 (initiation site) and STS98.4 (non-initiation site) were obtained and the ratio of these copy numbers represented the enrichment at initiation site. As a control, total MCF-7 DNA in the same size range was analyzed in parallel. Upon treatment with λ-exonuclease the enrichment factor remained the same (around 30 for the nascent DNA, and around 1 for the total DNA). (C) Nascent DNA enrichment at a known initiation site for DNA replication is maximal in synchronized MCF-7 cells entering into the S phase of the cell cycle. MCF-7 cells were arrested in the G1 phase of the cell cycle by keeping the cells for 48 hrs in estrogen-depleted medium. Upon transfer to a medium containing 10 nM estradiol aliquots were taken at times 0, 4, 8, 12, 14, and 17 hrs after estradiol addition. Aliquots were prepared for FACS and nascent DNA analysis. FACS analysis showed that cells entered the S-phase only after 10 hrs of estradiol addition (data not shown). Fraction#11 from the sucrose gradient containing DNA in the size range of [file pone.0017308.s001.tiff]

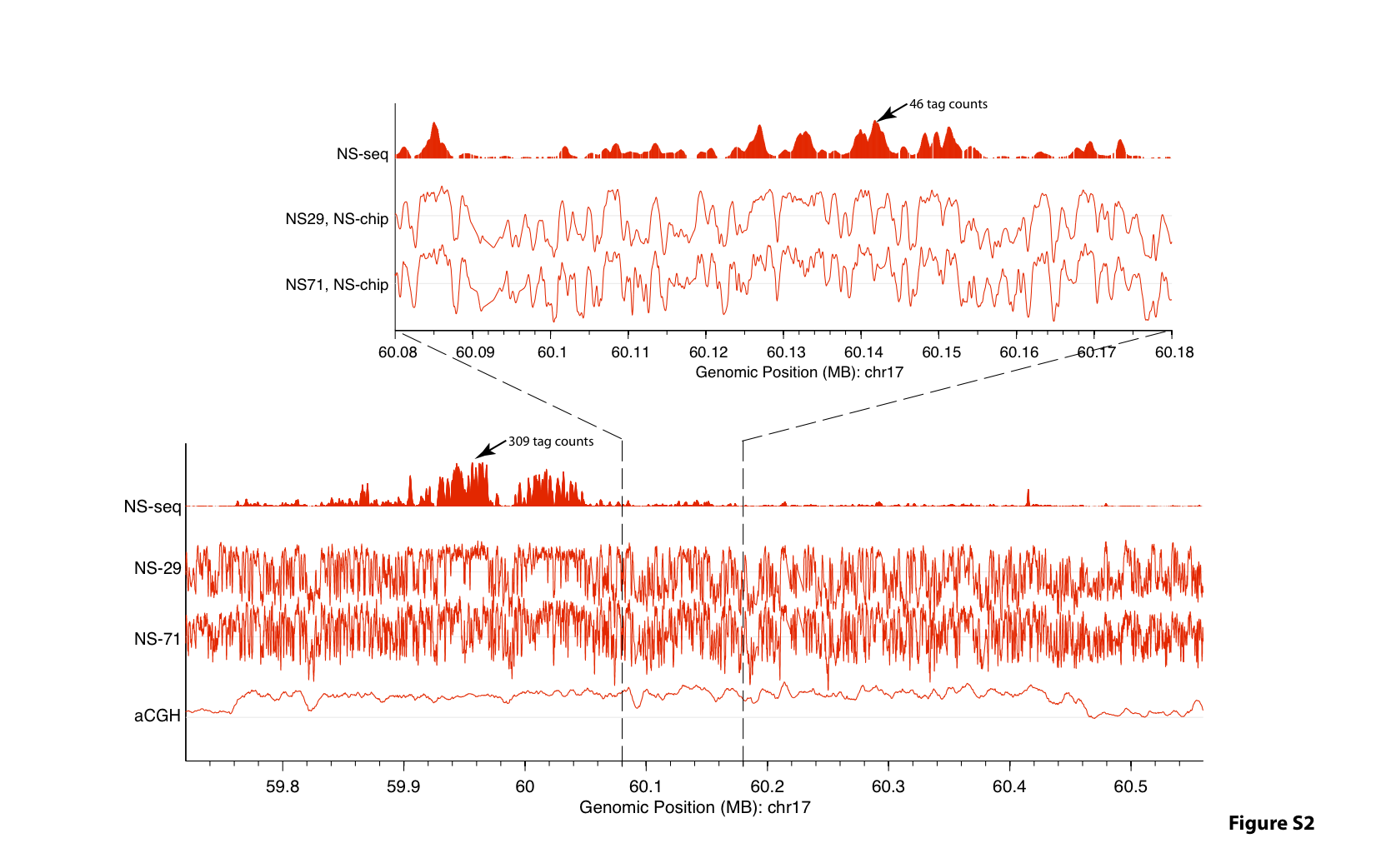

Supplement: Figure S2 — Correlation of NS-seq and NS-chip profiles at a low sequence tag region. Comparison of NS-seq data with two independent NS-chip data obtained from MCF-7 preparations along a chromosomal region containing a relatively low sequence tag abundance. (TIFF) [file pone.0017308.s002.tiff]

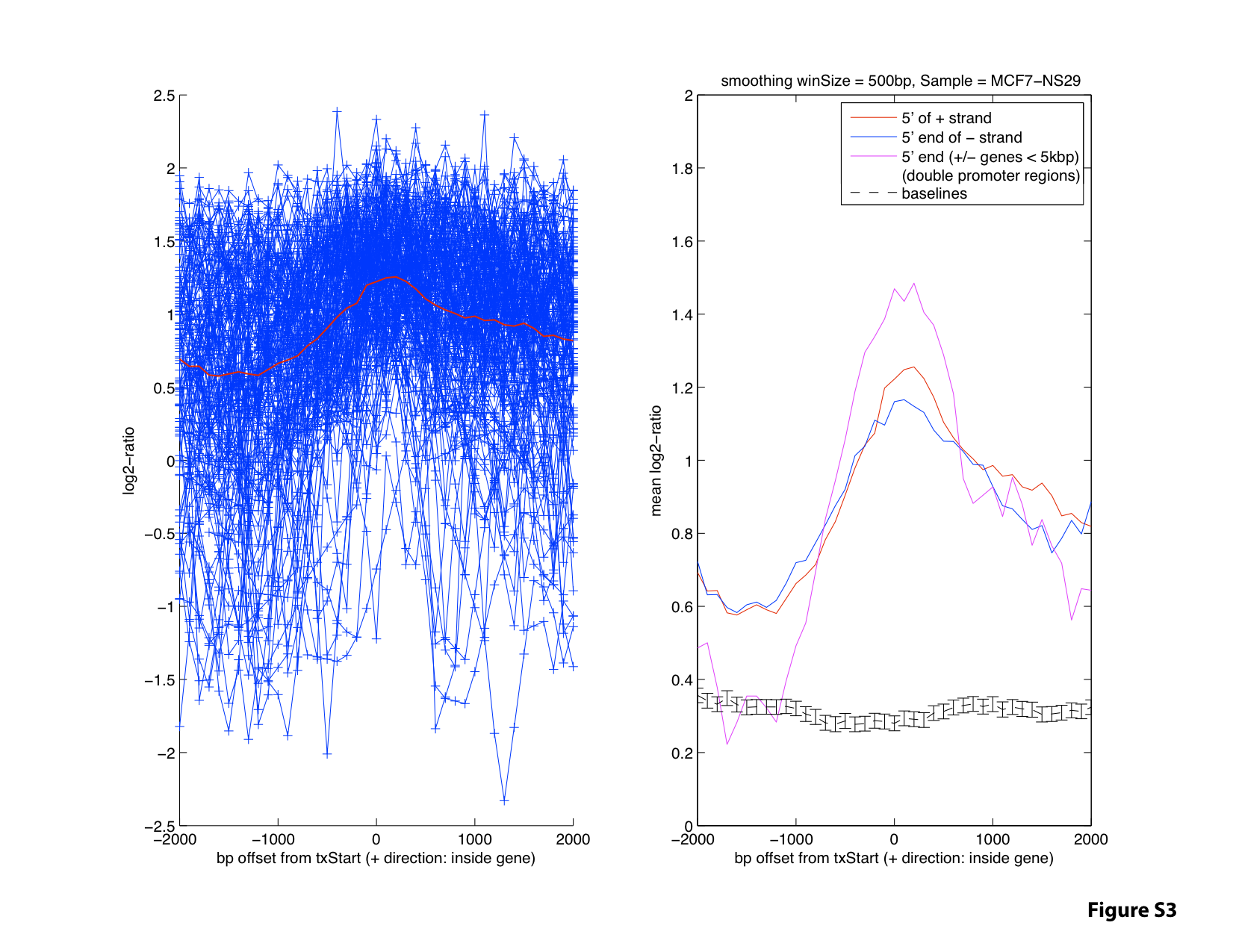

Supplement: Figure S3 — Enrichment of origin peaks at transcription start sites (TSSs) in MCF-7. A total of 334 TSSs on single promoters, and 21 TSSs in regions containing two diverging promoters were analyzed for origin peak enrichment using a smoothing window size of 500 bp. The left panel shows a composite plot for all genes centered at the TSS site. The right panel shows the enrichment profiles for genes transcribed in either orientation and for regions containing double promoters. Notice that the origin enrichment is more noticeable in this latter class. (TIFF) [file pone.0017308.s003.tiff]

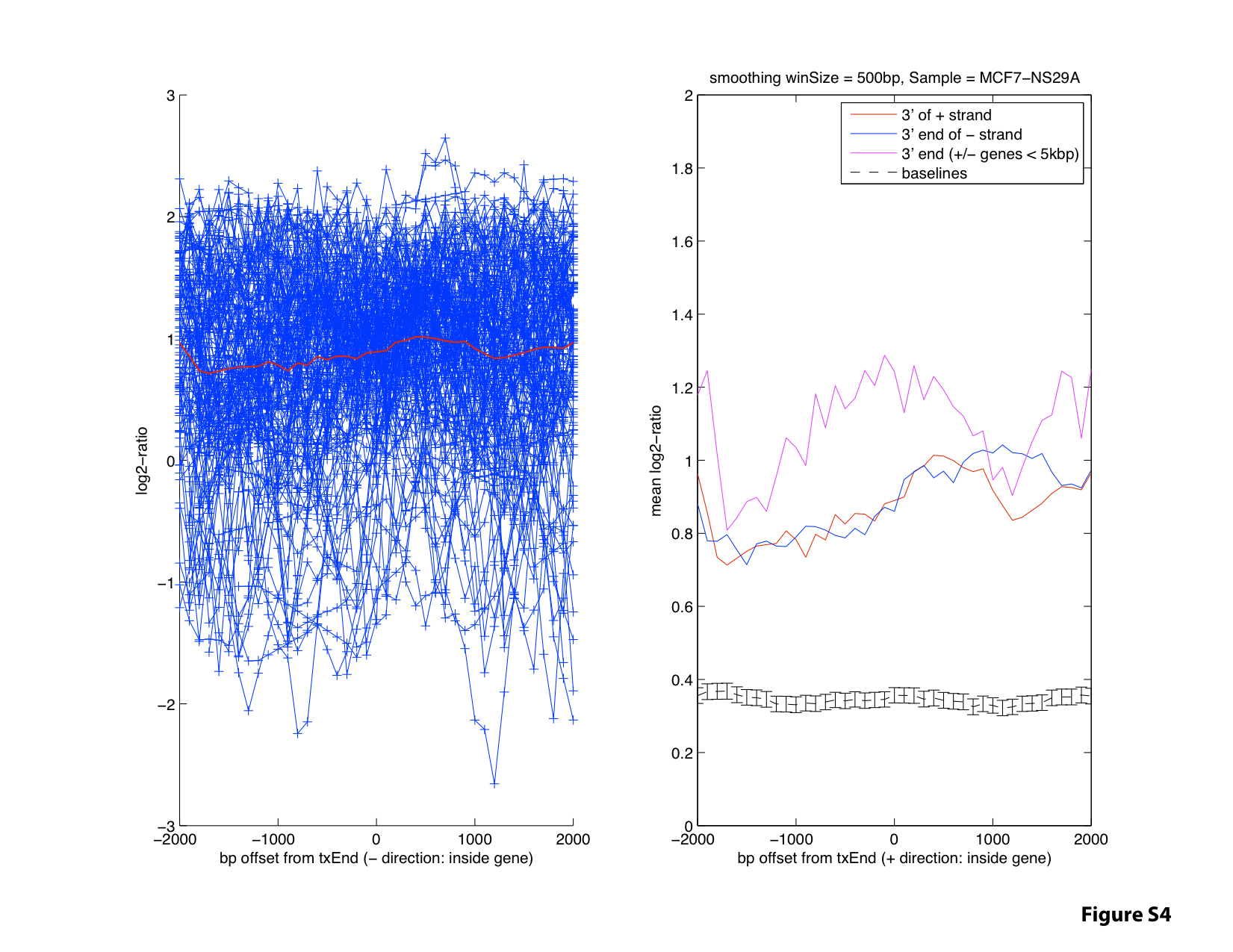

Supplement: Figure S4 — Origin peaks are not enriched at the 3′ ends of genes in MCF-7. A total of 316 unique txEnd sites, and 29 unique 3′–3′ sites (diverging transcripts within 5000 bp) present in the array were analyzed for origin peak enrichment using a smoothing window size of 500 bp. The left panel shows a composite plot for all genes centered around the txEnd site. The right panel shows the enrichment profiles for genes transcribed in either orientation and for regions containing double promoters. (TIFF) [file pone.0017308.s004.tiff]

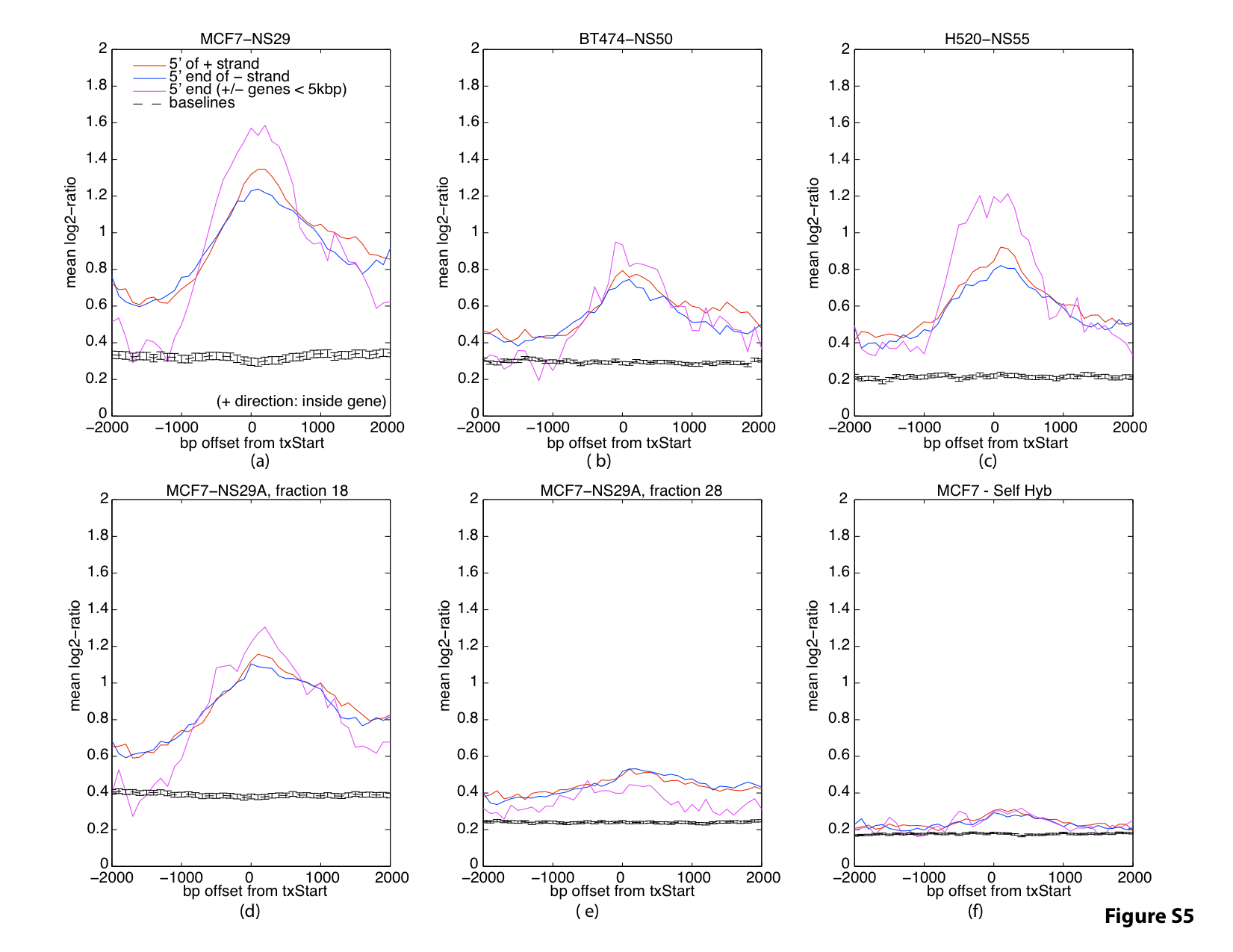

Supplement: Figure S5 — Enrichment of origin peaks at transcription start sites in all cell lines. Panels (a–c) show the enrichment of origin peaks at TSSs for short (0.7–1.5 kb) nascent DNA obtained from MCF-7, BT-474, and H520 cell lines , respectively. In each panel the enrichment at both single and regions containing two diverging promoters is indicated. Panels (d–e) show enrichment profiles for longer MCF-7 nascent DNA (1.5–3 kb, and >3 Kb, respectively). Panel f. Enrichment analysis on total sheared (0.5–1.5 kb) self- hybridized MCF-7 DNA is shown as a negative control. (TIFF) [file pone.0017308.s005.tiff]

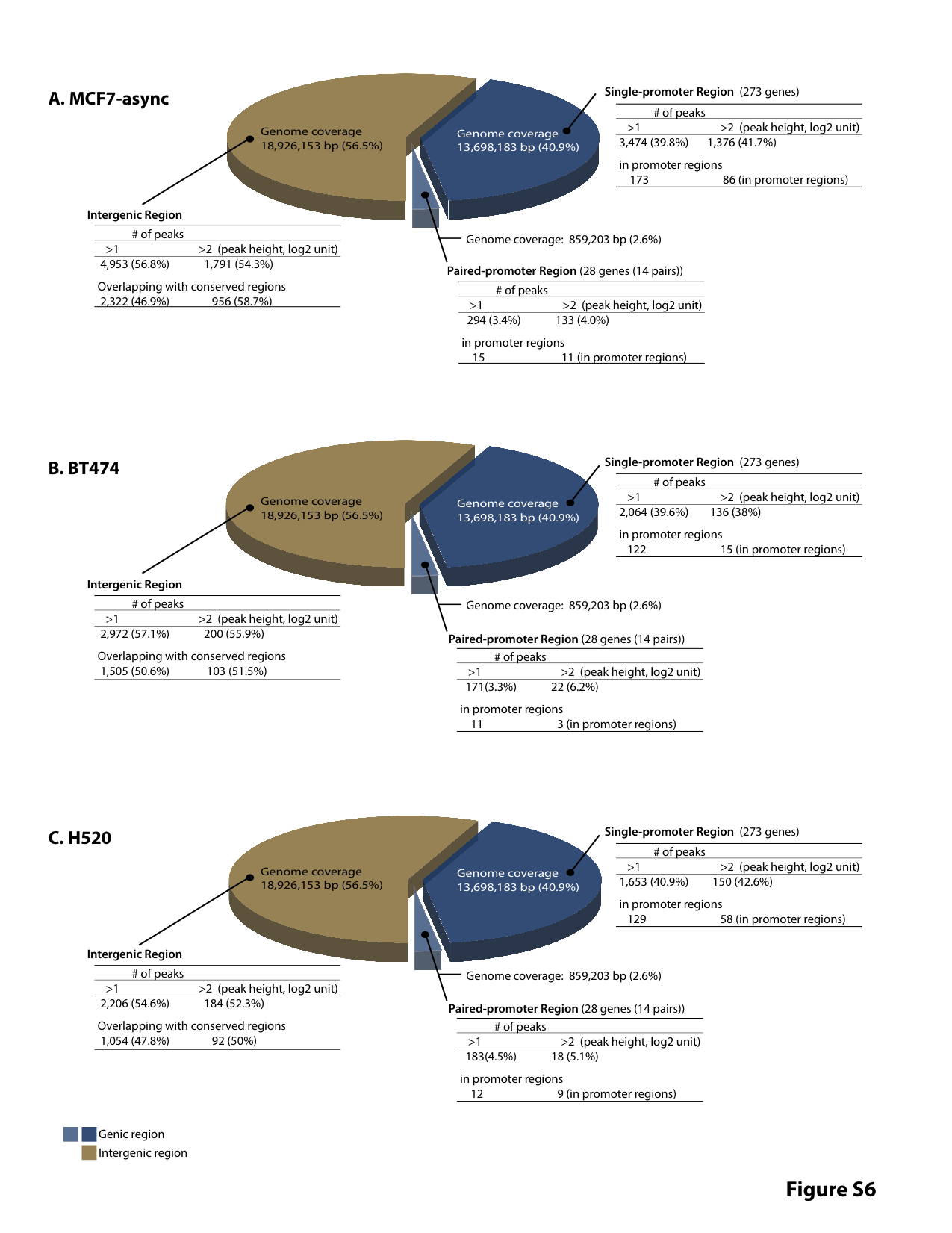

Supplement: Figure S6 — Summary of distribution of origin peaks among genic and intergenic regions and their association with promoters and conserved elements for all cell line used in the study (see Statistical Methods Supplement for detailed description of methods). Distribution of origins in asynchronous cultures of MCF-7, BT474, and H520 cancer lines, among both genic and intergenic regions (representing 43.5%, and 56.5% of all the sequences comprising the array, respectively). Genic regions have been further subdivided among those containing single promoters (dark blue pie segment), and those containing two closely space divergent promoters (light blue pie segment). The number of origins overlapping with evolutionarily conserved sequences in the intergenic region, is also indicated. (TIFF) [file pone.0017308.s006.tiff]

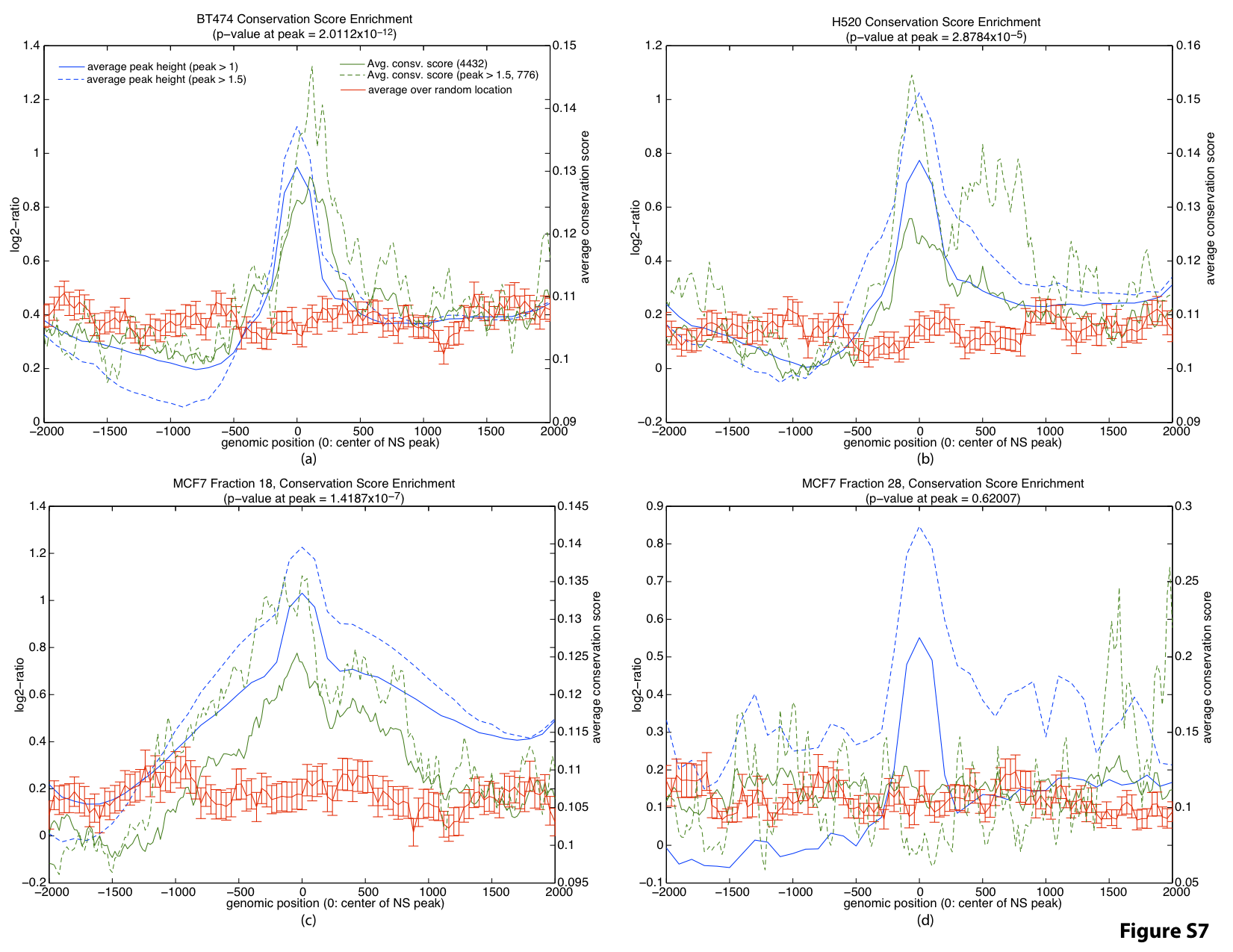

Supplement: Figure S7 — Association of origin enrichment with evolutionarily conservation scores in all cell lines. Panels (a–b) show the association of origin peaks with evolutionarily conservation scores for short (0.7–1.5 kb) nascent DNA obtained from BT-474, and H520 cell lines, respectively. Panels (c–d) show enrichment profiles for longer MCF-7 nascent DNA (fraction 18; 1.5–3 kb, and fraction 28; >3 Kb, respectively). (TIFF) [file pone.0017308.s007.tiff]

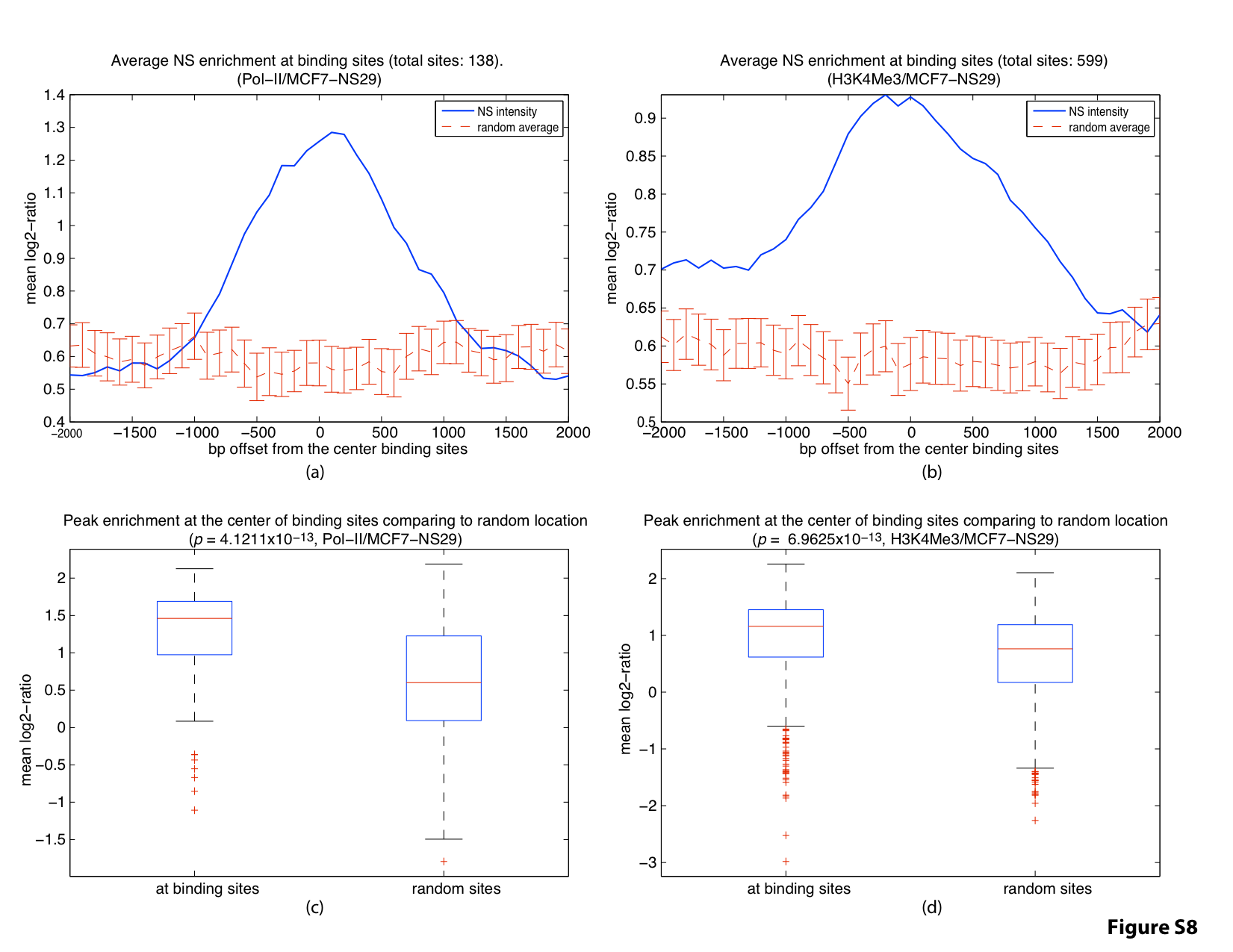

Supplement: Figure S8 — Association of origin enrichment with (a) Pol-II (blue line), and (b) H3K4Me3 (blue line) chromatin-binding sites in MCF-7, versus random locations (red line). Box-plot of enrichment level is shown in the bottom panels. (TIFF) [file pone.0017308.s008.tiff]

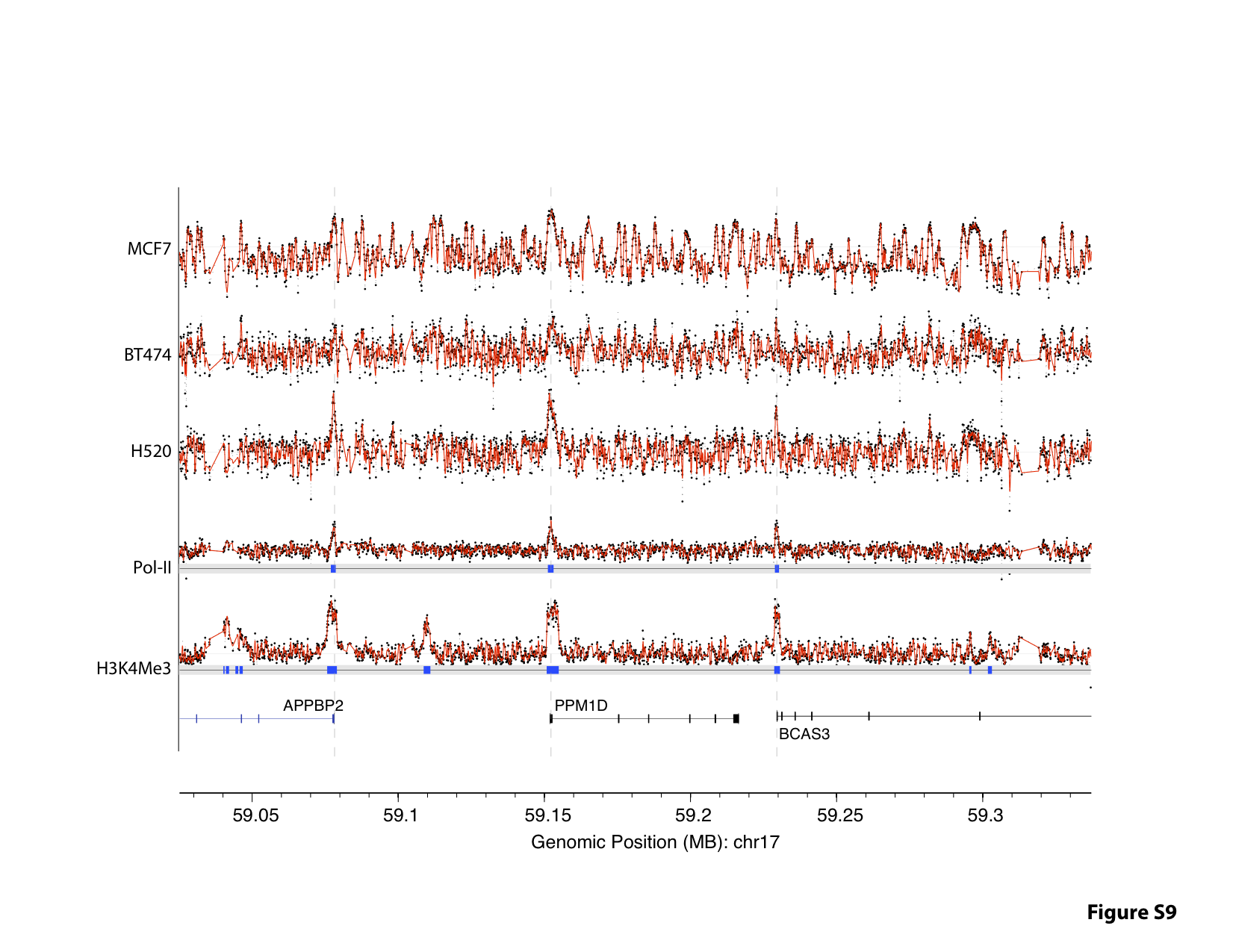

Supplement: Figure S9 — Correlation between efficiency of origin firing with Pol-II and H3K4Me3 binding along a 300 kb region of chr17. The distribution of origin peaks (peaks above the baseline indicated by a horizontal gray bar) obtained with MCF7, BT474, and H520 asynchronous cultures, along a 300 kb region of Chr17 (Chr17∶59,030,000–59,330,000) is shown. The position of RNA Pol-II and H3K4Me3 chromatin binding sites along this region as shown by ChIP on chip analysis is indicated by blue boxes. At the bottom of the figure, the position of three genes (APPBP2, PPMID, and BCAS3) present in this region is also illustrated. The vertical gray lines indicate the correlation between high origin peaks with the position of both Pol-II and H3K4Me3 binding sites. (TIFF) [file pone.0017308.s009.tiff]

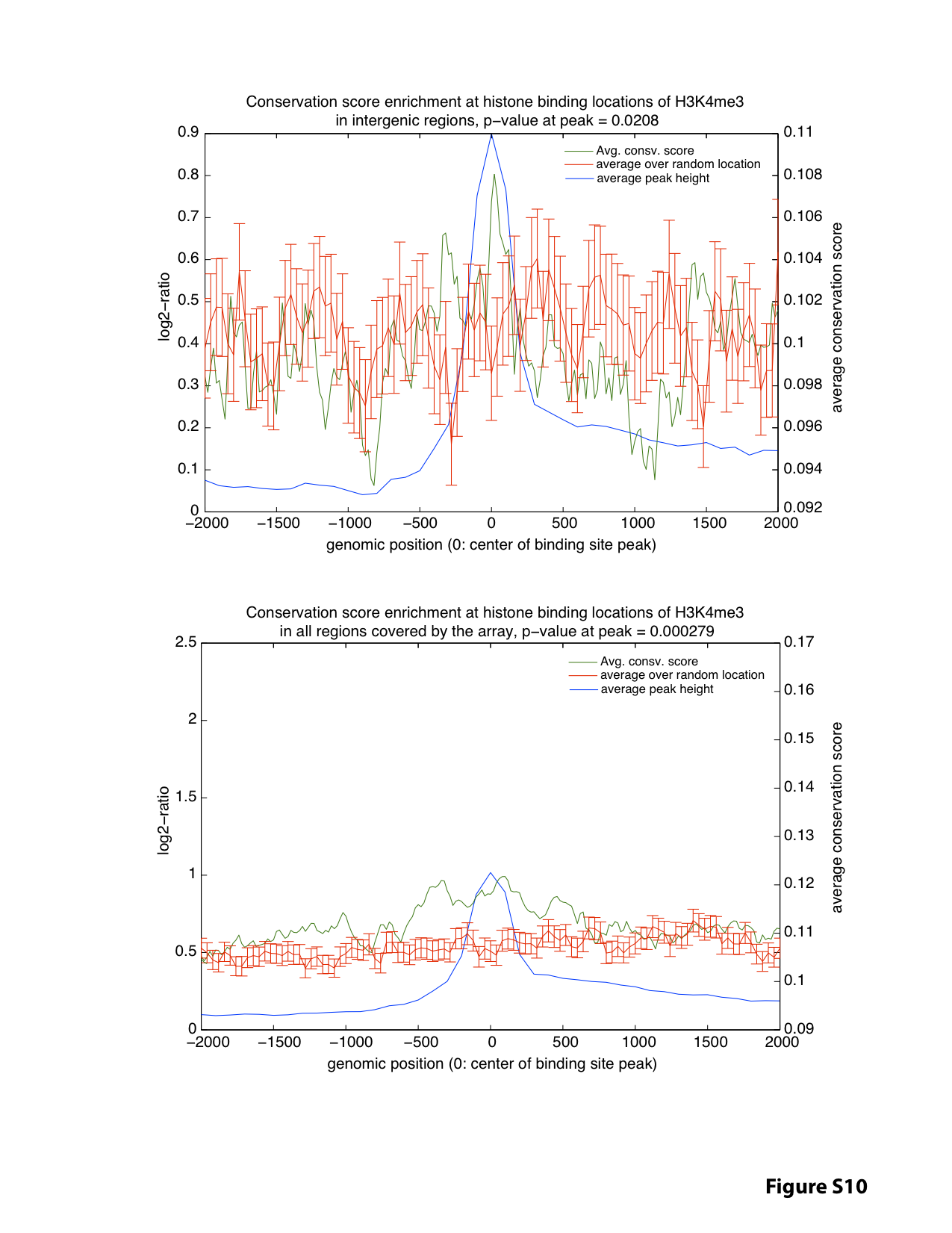

Supplement: Figure S10 — Conservation score enrichment at H3K4Me3 chromatin-binding sites. Top panel: Average conservation score at intergenic regions, around the center of H3K4Me3 binding site peak. Bottom panel: Conservation score for the entire region covered by the array. The random locations (equal to the number of corresponding binding sites) were sampled from the genome regions covered by the array, and the mean and standard error of log-ratios was plotted (red-colored lines) with error bars at 100 bp intervals. (TIFF) [file pone.0017308.s010.tiff]

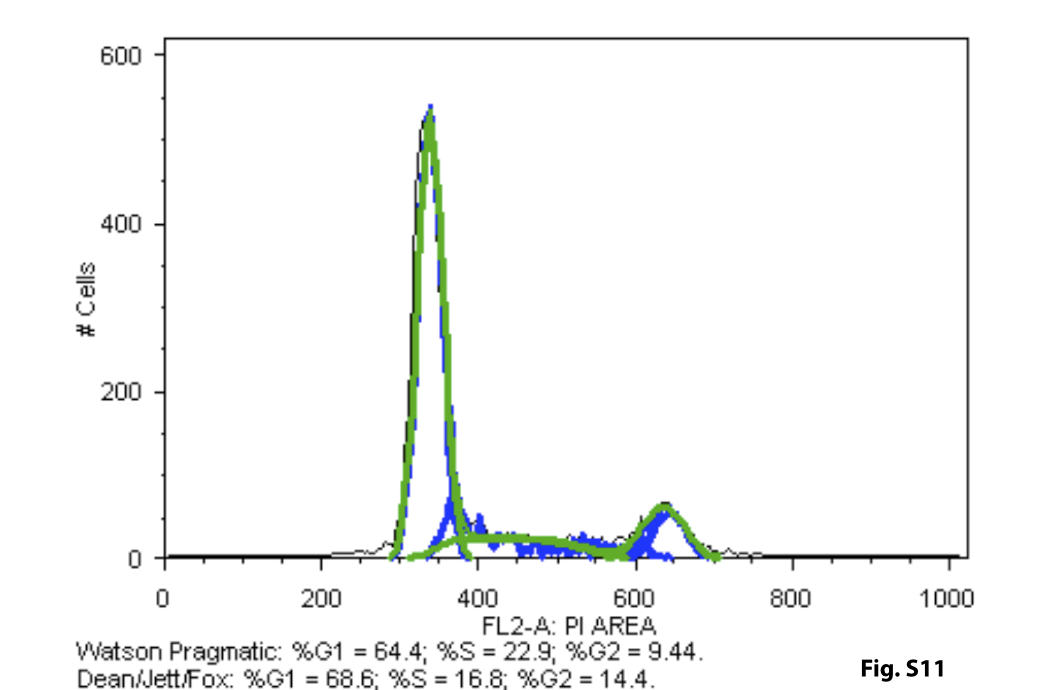

Supplement: Figure S11 — Profile of an exponential asynchronous culture of MCF-7 cells after fluorescent activated cell sorter (FACS) analysis. The distribution (percentage) of cells among the three major phases of the cell cycle (G1, S, and G2/M) is indicated for both Watson Pragmatic, and Dean/Jett/Fox methods of analysis. (TIFF) [file pone.0017308.s011.tiff]

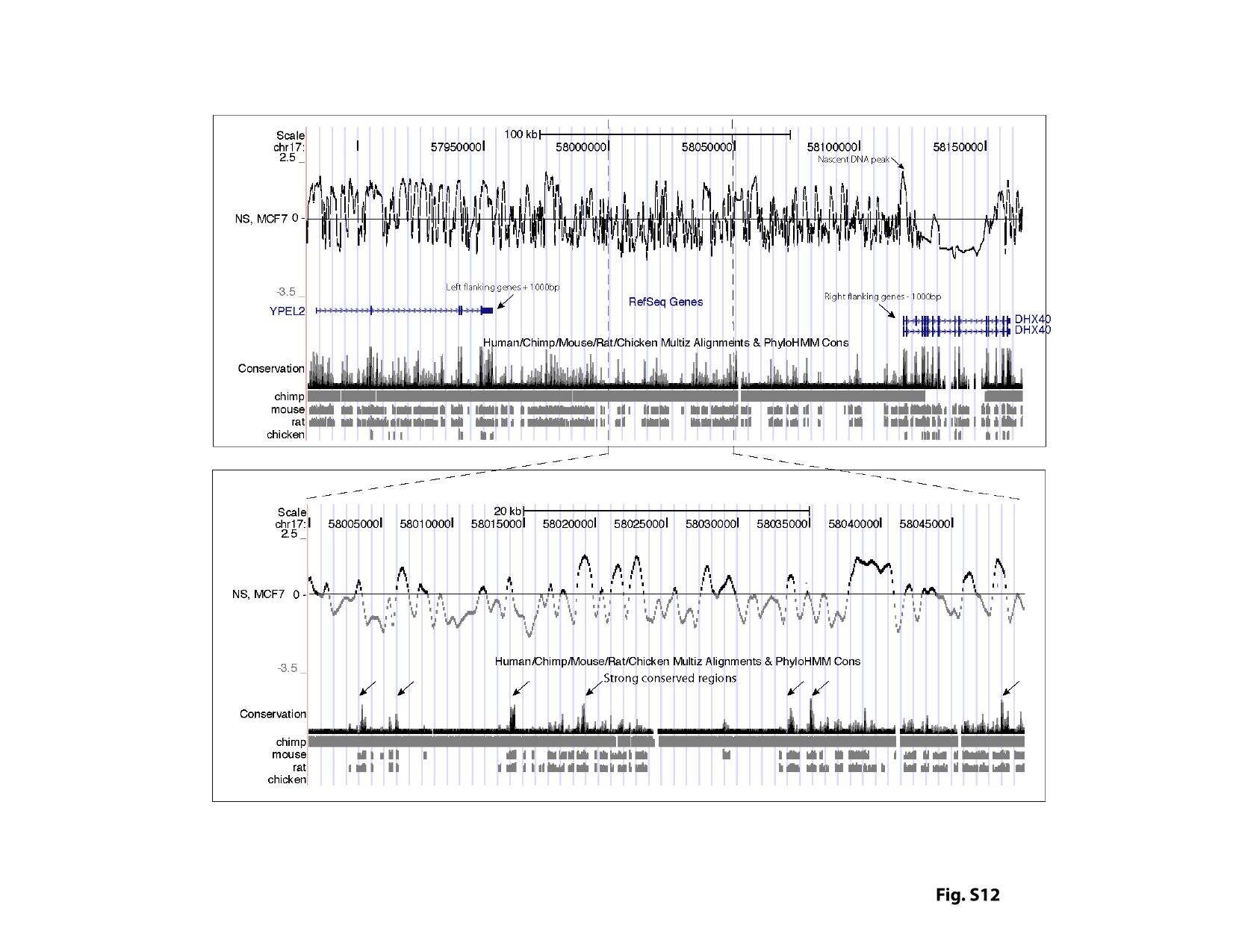

Supplement: Figure S12 — Diagrammatic representation of a 300 kb region of Chr17 containing non-genic evolutionary conserved elements. The top panel illustrates a 150 kb non-genic region flanked by the 3′-end of the YPEL2 gene and the 5′-end of the DHX40 gene. Bottom panel shows the location of DNA elements showing high conservation score among human, chimp, mouse, rat and chicken (indicated by arrows), within a 50 kb subregion (Chr17∶58,000,000–58,050,000). (TIFF) [file pone.0017308.s012.tiff]
