# Supplementary material for: Preferential Localization of Human Origins of DNA Replication at the 5′-Ends of Expressed Genes and at Evolutionarily Conserved DNA Sequences
Source: PLoS One. 2011 May 13;6(5):e17308. doi: 10.1371/journal.pone.0017308 (PMC3094316; doi:10.1371/journal.pone.0017308)
Supplement: Tables S1 — (PDF) [file pone.0017308.s013.pdf]

Table S1. Tiling array coverage (UCSC hg16 genome)

Chromosome	Genomic Position	Size (MB)	Region covered
chr2	210,973,598 - 211,173,420	0.2	RPE origin region
chr3	176,319,908 - 176,612,994	0.3	AC08180
	180,000,076 - 183,000,018	3	Normal region
	190,946,333 - 191,140,476	0.2	AC016966
chr8	128,567,328 - 128,815,284	0.2	c-myc origin region
chr11	4,688,730 - 5,790,319	1	beta-globin origin region ENm009
chr17	10,750,045 - 15,049,992	4.3	MCF7 normal region
	56,950,057 - 61,049,966	4.1	MCF7 High-gain region
chr19	2,329,235 - 2,445,509	0.1	Lamin B2 origin region
chr20	42,000,033 - 62,329,039	20.3	MCF7 gain region
**Total genomic coverage**		**33.5**	

Table S2. Validation Primer Pairs

Chrom	start	end	Notes	Strand	Left Primer	Right Primer
chr17	60019269	60019392	trough	+	AGCCCCTGGAGTAATCACCT	TGTCAGCTTCAGCTTCTGGA
chr17	60020455	60020679	p1	+	TCGCTTCTGAGTCATTGGTG	TTGGGTAGGAGTTGACTGGG
chr17	60021650	60021884	p2	+	CCCTTCTTGGCTTCTCAGTG	TGAGCTCTCAACTTGCCCTT
chr17	60022230	60022330	p3	+	CTGTCCTGTATGGCACATGG	TCTGCTGTGAAGGCTTTCCT
chr17	60023316	60023558	trough	+	GCAACCATGGCTACCAATTT	CCAAGATCACACCATTGCAC
chr17	60024230	60024380	trough	+	ATTTCCTCTCCTGCGAACTG	CAATCAAAAGCATCGGTGTG
chr17	60025225	60025291	trough	+	CTCCTGAAGGGGGTGGTAAT	AATAGCTCTGGGTCCCCACT
chr17	60026075	60026243	p4	+	GCTGACCCAAGTCCCTTACA	TGAGATCATTCGTGTCCAGC
chr17	60027449	60027556	trough	+	GATGATACCCAGGGCTTTGA	GCTGAGAGAACCCAGGACAG
chr17	60028604	60028769	p5	+	ATGGAGTCACCCAGCTCAAC	CCAGAGTGCCAAGAGAAAGG
chr17	60028838	60028951	trough	+	CGTCAGTACCAGGTCCCATC	AACCCTTCGGTTTCTGTCCT
chr17	60030072	60030153	trough	+	TGCTGACAGCTCACAATTCC	CCCTCCTTGATGTGACCAGT
chr17	60060940	60061152	trough	+	TGTTACCTGGGGGCAGATAC	CGGGCAACACTGCTAGACTC
chr17	60061644	60061821	trough	+	TTCCTAGCCTGTGTTTTGGG	CACAAATCCATTCCAAAGGG
chr17	60062496	60062586	trough	+	AGCTTCTTGCCACGTCAGAT	CTCACTGGTCACTGAGGCTG
chr17	60062928	60063099	p6	+	GCCTCCAGTTGGAAATTGAA	GCCTCTGTGAATGTGCAGAA
chr17	60063246	60063447	trough	+	TCCATTTTTGAGCACACAGC	GGCCCCAAAGTAAAGAAAGC
chr17	60064593	60064676	trough	+	TCAGAGCACCCCTCTAAGGA	AATCAGGACGTGGTTCAAGC
chr17	60066475	60066605	trough	+	CGAAGGACTGCAATCACAGA	CTCCTGGTCCGACTCTTTTG
chr17	60068988	60069169	trough	+	TGGGCCTCTGCATTAATTTC	GTCAGCAGACCTGTGTTGGA
chr17	60069823	60069935	trough	+	TGACTGAGAGTGGGCCTCAT	GCTGTTATCTGGCCTGGGTA
chr17	60070520	60070658	p7	+	ATCTGTGGACTGCAGAAGCA	CTTCGCTTCTCTGGTCAGGT
chr17	60072485	60072723	trough	+	TCCCAAAGTGCAGGGATTAC	GGCAACATTAATCCGGACAG
chr17	60074959	60075134	trough	+	GAAGCACCATCAAACTGGGT	CTCACAAAGTGCCTGGGATT
chr17	60076463	60076666	p8	+	AGCTGCTACTCTGGGCACAT	AAATTTGGTGCCGTGAGTTC
chr17	60077246	60077397	p9	+	AGACCAGGACTCCAAACGTG	AATCCAAAGCCCCAGAGATT
chr17	60078485	60078666	p10	+	AGAAGGGCAGACGTACAGGA	GCCAGACTCGGCAAATAAAA
chr17	60079458	60079552	trough	+	ACATGATGAAACCCCAGCTC	GCAACCTCTGTCTCTCTGGC
chr17	60081756	60081959	trough	+	TCTGCAAATCAGAGAGGGCT	TTTTGGCAGGACAAAGTTCC
chr17	60083280	60083533	trough	+	TCCGTTCACAGGAAATTCAA	TTCCAATCCGCTAGATGAGG
chr17	60084166	60084236	p11	+	TTGCAGTATCCCCTTAACCG	AGATGATTTCCCTCTCGGGT
chr17	60085224	60085301	trough	+	CACCCTAATGGGACGTGGTA	ATGGAATAGAGCCAAAGCGA
chr17	60086051	60086214	p12	+	CCTTCGTCCCAGACAATGTT	TGAACCTGTGAGAAGCAACG
chr17	60086929	60087094	trough	+	ATGGTTTGGGGGCTAAAAAG	CAGTGCATGAGAGTGCCAGT
chr17	60087340	60087506	trough	+	ACCTCAACAAGGGATTGGTT	TGACCATTTTCCCTAGCACA
chr17	60088854	60089057	p13	+	CCACACCCTTTCCAAGATGT	TGAAGATGTTTACTCCAGCATTG
chr17	60092094	60092192	trough	+	GCCTTGCCAGCATTTGTTAT	CAGCCAACAAGCGTATGAGA
chr17	60092502	60092662	trough	+	ATTCTTTGGCAAGGCTGATG	TGGGGAAAGGACTCCCTAGT

Gene	Known Origin Position (bp) Chromosome	Left - Right	Center	Nearest Peak	distance	Publication
c-myc	chr8	128,702,651-128,704,468	128,703,560		0*	Liu, *et al*, 2003
Beta-Globin	chr11	5,233,697 – 5,233,852	5,233,290	5,233,790	500	Aladjem, et al 1998
Lamin B2	chr19	2,379,022 – 2,379,307	2,379,165		0*	[Abdurashidova, et al, 2000] [Liu, et al, 2003]
RPE	chr2	211,085,356 – 211,086,497	211,085,927		0*	[Hu, et al, 2004]

* The center of origin is in the middle of a broad peak.

Table S3.

	Sample Name	total # of peaks	# of peaks (peak-to-trough ratio ≥ 1.0)	FDR (%)	Median distance (kbp)
1	MCF7-NS29	15,686	8,096	2.3%	3.05
2	MCF7-NS29A	16,800	9,083	3.1%	2.80
3	MCF7-Self Hyb	15,932	6	NA	2.6 Mbp
4	MCF7-E14NS24	18,018	10,245	2.4%	2.44
5	MCF7-E12NS23	14,256	5,103	< 1%	3.20
6	MCF7-NS29Afr18	13,665	3,724	< 1%	6.55
7	MCF7-NS29Afr28	13,893	192	< 1%	36.70
8	BT474-NS50	15,048	4,432	8%	4.57
9	H520-NS55	14,684	3,551	2.7%	3.80
10	H520-NS58	15,992	3,619	3.4%	3.60
11	MCF7 averaged	16,529	8,281	0.8%	2.95
12	H520 averaged	15,896	3,201	1%	3.87

Table S4.

Cell Line	***G1***	***S***	***G2/M***
MCF-7 Asynchronous	68.6 %	22.9%	9.4%
MCF-7 Asynchronous Lambda-exo	58%	37.6%	4.5%
MCF-7 Asynchronous Lambda-exo	73.6%	21.4%	5%
MCF-7 Synchronous 12 hr-release	70%	13.4%	5.9%
MCF-7 Synchronous 14hr-release	66.9%	15.8%	4.6%
BT-474 Asynchronous	55.1%	27%	11.8%
H520 Asynchronous	43.8%	37.2%	19.1%
H520 Asynchronous	38.4%	36.2%	25.5%

.

TableS5

Legends.

S1. Chromosomal coverage of custom array DNA used in our study (based on hg16 genome). Copy number features in the regions covered refer to the MCF-7 cell line only.

S2. Primer sets used to validate array profiles by real time PCR.

S3. Concordance of array positioning of origin peaks with the location of four previously described origins of DNA replication (c-myc, β-globin, Lamin B2, and RPE respectively).

S4. Mean peak-to-peak distance for all cell lines used in this study, at peak to trough height of at least 1.0 (log2-ratio) and a Savitzky-Golay smoothing window size of 500 bp (see Statistical Methods Supplement). NA in the FDR column means that no peaks were detected, while FDR denoted with the (<) sign indicates than an insufficient count of peaks (less than 10) were detected from random simulation. Samples 11 and 12 represent averaged results reported in the main text.

S5. Percentage distribution in each cell cycle phase (G1, S, and G2/M) in asynchronous cultures of all cancer cell lines used in this study.

Table S1. Tiling array coverage (UCSC hg16 genome)

Chromosome	Genomic Position	Size (MB)	Region covered
chr2	210,973,598 - 211,173,420	0.2	RPE origin region
chr3	176,319,908 - 176,612,994	0.3	AC08180
	180,000,076 - 183,000,018	3	Normal region
	190,946,333 - 191,140,476	0.2	AC016966
chr8	128,567,328 - 128,815,284	0.2	c-myc origin region
chr11	4,688,730 - 5,790,319	1	beta-globin origin region ENm009
chr17	10,750,045 - 15,049,992	4.3	MCF7 normal region
	56,950,057 - 61,049,966	4.1	MCF7 High-gain region
chr19	2,329,235 - 2,445,509	0.1	Lamin B2 origin region
chr20	42,000,033 - 62,329,039	20.3	MCF7 gain region
**Total genomic coverage**		**33.5**	

Table S2. Validation Primer Pairs

Chrom	start	end	Notes	Strand	Left Primer	Right Primer
chr17	60019269	60019392	trough	+	AGCCCCTGGAGTAATCACCT	TGTCAGCTTCAGCTTCTGGA
chr17	60020455	60020679	p1	+	TCGCTTCTGAGTCATTGGTG	TTGGGTAGGAGTTGACTGGG
chr17	60021650	60021884	p2	+	CCCTTCTTGGCTTCTCAGTG	TGAGCTCTCAACTTGCCCTT
chr17	60022230	60022330	p3	+	CTGTCCTGTATGGCACATGG	TCTGCTGTGAAGGCTTTCCT
chr17	60023316	60023558	trough	+	GCAACCATGGCTACCAATTT	CCAAGATCACACCATTGCAC
chr17	60024230	60024380	trough	+	ATTTCCTCTCCTGCGAACTG	CAATCAAAAGCATCGGTGTG
chr17	60025225	60025291	trough	+	CTCCTGAAGGGGGTGGTAAT	AATAGCTCTGGGTCCCCACT
chr17	60026075	60026243	p4	+	GCTGACCCAAGTCCCTTACA	TGAGATCATTCGTGTCCAGC
chr17	60027449	60027556	trough	+	GATGATACCCAGGGCTTTGA	GCTGAGAGAACCCAGGACAG
chr17	60028604	60028769	p5	+	ATGGAGTCACCCAGCTCAAC	CCAGAGTGCCAAGAGAAAGG
chr17	60028838	60028951	trough	+	CGTCAGTACCAGGTCCCATC	AACCCTTCGGTTTCTGTCCT
chr17	60030072	60030153	trough	+	TGCTGACAGCTCACAATTCC	CCCTCCTTGATGTGACCAGT
chr17	60060940	60061152	trough	+	TGTTACCTGGGGGCAGATAC	CGGGCAACACTGCTAGACTC
chr17	60061644	60061821	trough	+	TTCCTAGCCTGTGTTTTGGG	CACAAATCCATTCCAAAGGG
chr17	60062496	60062586	trough	+	AGCTTCTTGCCACGTCAGAT	CTCACTGGTCACTGAGGCTG
chr17	60062928	60063099	p6	+	GCCTCCAGTTGGAAATTGAA	GCCTCTGTGAATGTGCAGAA
chr17	60063246	60063447	trough	+	TCCATTTTTGAGCACACAGC	GGCCCCAAAGTAAAGAAAGC
chr17	60064593	60064676	trough	+	TCAGAGCACCCCTCTAAGGA	AATCAGGACGTGGTTCAAGC
chr17	60066475	60066605	trough	+	CGAAGGACTGCAATCACAGA	CTCCTGGTCCGACTCTTTTG
chr17	60068988	60069169	trough	+	TGGGCCTCTGCATTAATTTC	GTCAGCAGACCTGTGTTGGA
chr17	60069823	60069935	trough	+	TGACTGAGAGTGGGCCTCAT	GCTGTTATCTGGCCTGGGTA
chr17	60070520	60070658	p7	+	ATCTGTGGACTGCAGAAGCA	CTTCGCTTCTCTGGTCAGGT
chr17	60072485	60072723	trough	+	TCCCAAAGTGCAGGGATTAC	GGCAACATTAATCCGGACAG
chr17	60074959	60075134	trough	+	GAAGCACCATCAAACTGGGT	CTCACAAAGTGCCTGGGATT
chr17	60076463	60076666	p8	+	AGCTGCTACTCTGGGCACAT	AAATTTGGTGCCGTGAGTTC
chr17	60077246	60077397	p9	+	AGACCAGGACTCCAAACGTG	AATCCAAAGCCCCAGAGATT
chr17	60078485	60078666	p10	+	AGAAGGGCAGACGTACAGGA	GCCAGACTCGGCAAATAAAA
chr17	60079458	60079552	trough	+	ACATGATGAAACCCCAGCTC	GCAACCTCTGTCTCTCTGGC
chr17	60081756	60081959	trough	+	TCTGCAAATCAGAGAGGGCT	TTTTGGCAGGACAAAGTTCC
chr17	60083280	60083533	trough	+	TCCGTTCACAGGAAATTCAA	TTCCAATCCGCTAGATGAGG
chr17	60084166	60084236	p11	+	TTGCAGTATCCCCTTAACCG	AGATGATTTCCCTCTCGGGT
chr17	60085224	60085301	trough	+	CACCCTAATGGGACGTGGTA	ATGGAATAGAGCCAAAGCGA
chr17	60086051	60086214	p12	+	CCTTCGTCCCAGACAATGTT	TGAACCTGTGAGAAGCAACG
chr17	60086929	60087094	trough	+	ATGGTTTGGGGGCTAAAAAG	CAGTGCATGAGAGTGCCAGT
chr17	60087340	60087506	trough	+	ACCTCAACAAGGGATTGGTT	TGACCATTTTCCCTAGCACA
chr17	60088854	60089057	p13	+	CCACACCCTTTCCAAGATGT	TGAAGATGTTTACTCCAGCATTG
chr17	60092094	60092192	trough	+	GCCTTGCCAGCATTTGTTAT	CAGCCAACAAGCGTATGAGA
chr17	60092502	60092662	trough	+	ATTCTTTGGCAAGGCTGATG	TGGGGAAAGGACTCCCTAGT

	Known Origin Position (bp)					
Gene	Chromosome	Left - Right	Center	Nearest Peak	distance	Publication
c-myc	chr8	128,702,651-128,704,468	128,703,560		0*	Liu, *et al*, 2003
Beta-Globin	chr11	5,233,697 – 5,233,852	5,233,290	5,233,790	500	Aladjem, et al 1998
Lamin B2	chr19	2,379,022 – 2,379,307	2,379,165		0*	[Abdurashidova, et al, 2000] [Liu, et al, 2003]
RPE	chr2	211,085,356 – 211,086,497	211,085,927		0*	[Hu, et al, 2004]

* The center of origin is in the middle of a broad peak.

Table S3.

	Sample Name	total # of peaks	# of peaks (peak-to-trough ratio ≥ 1.0)	FDR (%)	Median distance (kbp)
1	MCF7-NS29	15,686	8,096	2.3%	3.05
2	MCF7-NS29A	16,800	9,083	3.1%	2.80
3	MCF7-Self Hyb	15,932	6	NA	2.6 Mbp
4	MCF7-E14NS24	18,018	10,245	2.4%	2.44
5	MCF7-E12NS23	14,256	5,103	< 1%	3.20
6	MCF7-NS29Afr18	13,665	3,724	< 1%	6.55
7	MCF7-NS29Afr28	13,893	192	< 1%	36.70
8	BT474-NS50	15,048	4,432	8%	4.57
9	H520-NS55	14,684	3,551	2.7%	3.80
10	H520-NS58	15,992	3,619	3.4%	3.60
11	MCF7 averaged	16,529	8,281	0.8%	2.95
12	H520 averaged	15,896	3,201	1%	3.87

Table S4.
